# Hepatocellular Carcinoma LINC01116 Outcompetes T Cells for Linoleic Acid and Accelerates Tumor Progression

**DOI:** 10.1002/advs.202400676

**Published:** 2024-03-09

**Authors:** Kun Ma, Junhui Chu, Yufeng Liu, Linmao Sun, Shuo Zhou, Xianying Li, Changyong Ji, Ning Zhang, Xinyu Guo, Shuhang Liang, Tianming Cui, Qingsong Hu, Jiabei Wang, Yao Liu, Lianxin Liu

**Affiliations:** ^1^ Department of General Surgery Key Laboratory of Hepatosplenic Surgery Ministry of Education The First Affiliated Hospital of Harbin Medical University Harbin 150001 China; ^2^ Department of Hepatobiliary Surgery Centre for Leading Medicine and Advanced Technologies of IHM The First Affiliated Hospital of USTC Division of Life Sciences and Medicine University of Science and Technology of China Hefei Anhui 230001 China; ^3^ Anhui Province Key Laboratory of Hepatopancreatobiliary Surgery Hefei Anhui 230001 China; ^4^ Anhui Provincial Clinical Research Center for Hepatobiliary Diseases Hefei Anhui 230001 China; ^5^ Department of Gastrointestinal Surgery Anhui Province Key Laboratory of Hepatopancreatobiliary Surgery The First Affiliated Hospital of USTC Division of Life Sciences and Medicine University of Science and Technology of China Hefei Anhui 230001 China

**Keywords:** EWSR1, hepatocellular carcinoma, immunotherapy, LINC01116, lipid metabolism

## Abstract

Hepatocellular carcinoma (HCC) is the most common type of primary liver cancer with a highly immunosuppressive tumor microenvironment and a typical pattern of disturbances in hepatic lipid metabolism. Long non‐coding RNAs are shown to play an important role in the regulation of gene expression, but much remains unknown between tumor microenvironment and lipid metabolism as a bridging molecule. Here, long intergenic nonprotein coding RNA 01116 (LINC01116) acts as this molecular which is frequently upregulated in HCC patients and associated with HCC progression in vitro and in vivo is identified. Mechanistically, LINC01116 stabilizes EWS RNA‐binding protein 1 (EWSR1) by preventing RAD18 E3 Ubiquitin Protein Ligase (RAD18) ‐mediated ubiquitination. The enhanced EWSR1 protein upregulates peroxisome proliferator activated receptor alpha (PPARA) and fatty acid binding protein1 (FABP1) expression, a long‐chain fatty acid (LCFA) transporter, and thus cancer cells outcompete T cells for LCFAs, especially linoleic acid, for seeding their own growth, leading to T cell malfunction and HCC malignant progression. In a preclinical animal model, the blockade of LINC01116 leads to enhanced efficacy of anti‐PD1 treatment accompanied by increased cytotoxic T cell and decreased exhausted T cell infiltration. Collectively, LINC01116 is an immunometabolic lncRNA and the LINC01116‐EWSR1‐PPARA‐FABP1 axis may be targetable for cancer immunotherapy.

## Graphical abstract

LINC01116, an immunometabolic lncRNA, promoted its own growth through competitive consumption of linoleic acid and attenuated the efficacy of immunotherapy is identified.

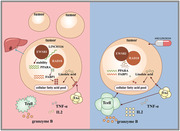

## 1 Introduction

Hepatocellular carcinoma (HCC) is the sixth most common cancer and the third most common cause of cancer‐related death.^[^ ^1^ ^]^ HCC is a primary tumor caused by an increase in genomic mutational load in hepatocytes in response to multiple risk stimuli, and its occurrence may be associated with hepatitis B and C virus infection, alcohol consumption, and obesity.^[^ ^2^ ^]^ Although there are many treatment options for HCC, due to the inconspicuous early symptoms, the rapid proliferation of cancer cells, and the rich blood supply of the liver, the development is extremely rapid, and metastatic lesions are easily formed.^[^ ^3^ ^]^ The treatment efficacy is not ideal, and the overall survival rate and disease‐free survival rate have not substantially improved. Therefore, it is important to analyze the underlying mechanisms of HCC proliferation and metastasis to identify therapeutic targets and prognostic markers.

Reprogramming of lipid metabolism is one of the most prominent metabolic alterations in cancer and includes alterations in fatty acid (FA) transport, de novo FA synthesis, lipid droplet storage, and ATP production via FA oxidation.^[^ ^4^ ^]^ Enhanced lipid synthesis or uptake contributes to the rapid growth of cancer cells and tumor formation. In tumor tissues, lipids ensure the structural integrity of biofilms, provide energy, influence the regulation of redox homeostasis, promote plasticity, angiogenesis, and microenvironment remodeling, mediate the regulation of inflammatory responses, and function as signal transductors, suggesting that lipids influence a myriad of processes related to tumorigenesis and progression.^[^ ^5^ ^]^ Therefore, targeting genes involved in lipid metabolism may be a promising therapeutic approach.

The tumor microenvironment (TME) is composed of immune cells, tumor cells, and inflammatory factors. Due to the vigorous metabolism of tumor cells, they usually produce some metabolites that are unfavorable to immune cells, and they also change a variety of carrier proteins and deprive a large number of nutrients.^[^ ^6^ ^]^ These characteristics underlie tumor insensitivity to immunotherapy. In the microenvironment, the availability of nutrients and energy to immune cells is a primary regulator of immune cell metabolism. As part of the adaptive and innate immune response, fatty acid oxidation plays a critical role. Fatty acid oxidation has been found to have a key role in macrophage differentiation, regulating T cell responses, and the generation and maintenance of long‐lived memory CD8^+^ T cells. Exogenous free fatty acids and oxidative metabolism enable tissue‐resident memory T cells to persist in tissues and mediate protective immunity.^[^ ^7^, ^8^, ^9^ ^]^ Polyunsaturated fatty acids improve anti‐inflammatory cytokine production^[^ ^10^ ^]^ and short‐chain fatty acids augment CD8^+^ T cell effector function.^[^ ^11^, ^12^, ^13^ ^]^ Lipids stored in dendritic cells (DCs) play a key role in their immunogenicity. Adipogenesis and endoplasmic reticulum stress are associated with lipid‐rich DC immunogenicity. The acidic TME can also lead to lipid droplet accumulation in DCs, which leads to DC dysfunction.^[^ ^14^ ^]^ DCs accumulate lipid droplets mostly via CD36‐mediated uptake of exogenous lipids.^[^ ^15^ ^]^ DC function may be improved by targeting lipidic transporters selectively. Clinically, postoperative lipid accumulation caused by upregulated CD36 and MSR1 expression can impair NK cell cytotoxicity and tumor control. M2 macrophage polarization is driven by SLC27A1 overexpression by enhancing long‐chain acyl‐CoA synthetase activity and fatty acid uptake.^[^ ^16^ ^]^ Thus, restoring the function of immune cells by metabolic interventions is a means of sensitizing tumors to immunotherapy.

LINC01116 is a long noncoding RNA, and its abnormal expression is associated with a variety of cancers. LINC01116 promotes the proliferation, migration, and invasion of lung cancer, gastric cancer, and colorectal cancer cells.^[^ ^17^, ^18^, ^19^, ^20^ ^]^ Although the role of LINC01116 in tumor cell proliferation and metastasis has been extensively studied, its role in the tumor immune microenvironment and metabolic reprogramming is still unknown. Elucidating its role may help to resolve the challenge of drug resistance and enhance therapeutic efficacy.

Here, we identify LINC01116 by analyzing The Cancer Genome Atlas (TCGA) database, a Gene Set Enrichment Analysis (GSEA) dataset, and the ImmPort database and find that its expression rises with HCC grade. Moreover, LINC01116 promotes tumor proliferation and metastasis by increasing lipid uptake. LINC01116 stabilizes EWSR1 expression by blocking the binding of EWSR1 to RAD18, which promotes the PPARA/FABP1 signaling axis, thereby depriving T cells of required linoleic acid and inactivating T cells. Pharmacological inhibition of LINC01116 can promote the efficacy of immunotherapy. These results suggest that LINC01116 can be a promising therapeutic target.

## 2 Results

### 2.1 LINC01116 is Highly Expressed in HCC Patients and Associated with HCC Progression

**Figure 1:**
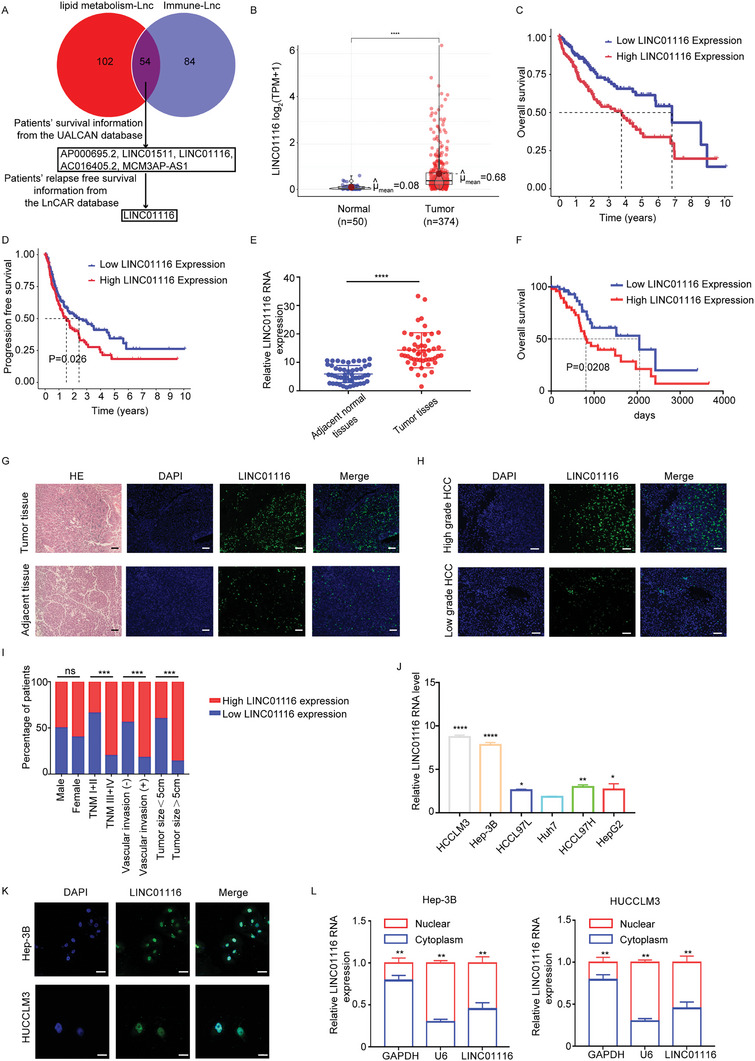
LINC01116 is highly expressed in HCC patients and associated with HCC progression. A) TCGA database, ImmPort database, and a GSEA dataset were used to screen out the target long noncoding RNAs. B) Relative expression of LINC01116 in normal liver and HCC specimens from TCGA database. C) Kaplan‐Meier plot of overall survival stratified by LINC01116‐high/low status. Log‐rank Mantel‐Cox test. D) Kaplan‐Meier plot of progression‐free survival of HCC patients in each group in TCGA. Log‐rank Mantel‐Cox test. E) Relative expression of LINC01116 in 50 pairs of tumor tissues and adjacent tissues measured by qRT‐PCR. F) Kaplan‐Meier plot of overall survival of HCC patients in (E). Log‐rank Mantel‐Cox test. G) Representative images of FISH assays (scale bars 20 µm) and hematoxylin and eosin staining (scale bars, 100 µm) in tumor and adjacent tissues. H) FISH assays showed LINC01116 expression in low‐grade and high‐grade HCC patients. Scale bars 20 µm. I) Percentage of patients with relatively high expression and low expression of LINC01116 according to the clinical parameters. J) The relative expression of LINC01116 in the indicated HCC cell lines. K) FISH assays showed the cellular localization of LINC01116 in HCC cells. Scale bars 20 µm. L) Nuclear‐cytoplasmic fractionation assays showed that LINC01116 was mostly localized in the nucleus of HCC cells. The data are presented as the mean± SD. ^*^ *p* < 0.05; ^**^ *p* < 0.01; ^***^ *p* < 0.001; ^****^ *p* < 0.0001.

To identify unknown molecules that modulate tumor microenvironment by lipid metabolic reprograming, we obtained a lipid metabolism‐related gene set from the GSEA database, an immune gene set from the ImmPort database, and a lncRNA expression matrix from the TCGA liver cancer database. Then, we performed differential expression analysis of the above‐obtained lncRNAs in tumor tissues and normal tissues. Based on a correlation test, lncRNAs related to immune genes and lipid metabolism genes were screened. Finally, we obtained 54 lncRNAs. Considering the value of biological molecules in clinical diagnosis, we found that only LINC01116 was associated with the prognosis of patients (**Figure** **1**A; Figure S1A, Supporting Information). Meanwhile, the conservation of LINC01116 was analyzed (Figure S1B, Supporting Information). Therefore, LINC01116 was selected as our following study subject. The analysis of HCC data in the TCGA database showed that LINC01116 was highly expressed in tumor tissues (Figure 1B). Moreover, the LINC01116‐high cluster of HCC patients displayed strikingly inferior overall and progression‐free survival, and a higher tumor grade (Figure 1C,D; Figure S1C, Supporting Information). To further verify LINC01116 expression in HCC patients, qRT‐PCR and FISH were performed on our HCC database. Combined with the clinicopathological characterization and statistical analysis, we confirmed that LINC01116 was highly expressed in tumor tissues and was associated with vascular invasion, tumor node metastasis (TNM) stage, tumor size, and overall survival (Figure 1E–I). Interestingly, we found that in six hepatoma cell lines, LINC01116 expression was highest in HCCLM3 cells and Hep‐3B cells, which have strong invasive ability, and LINC01116 expression was the lowest in less invasive Huh‐7 cells, which was in agreement with our histopathological studies (Figure 1J). The functions of lncRNAs are mostly related to their intracellular localization. We then performed nuclear‐cytoplasmic fractionation experiments and FISH assays. The results showed that LINC01116 was mostly localized to the nucleus (Figure 1K–L). According to these data, LINC01116 is highly expressed in HCC patients and correlated with HCC progression.

### 2.2 LINC01116 Promotes HCC Progression in Vitro and in Vivo

**Figure 2:**
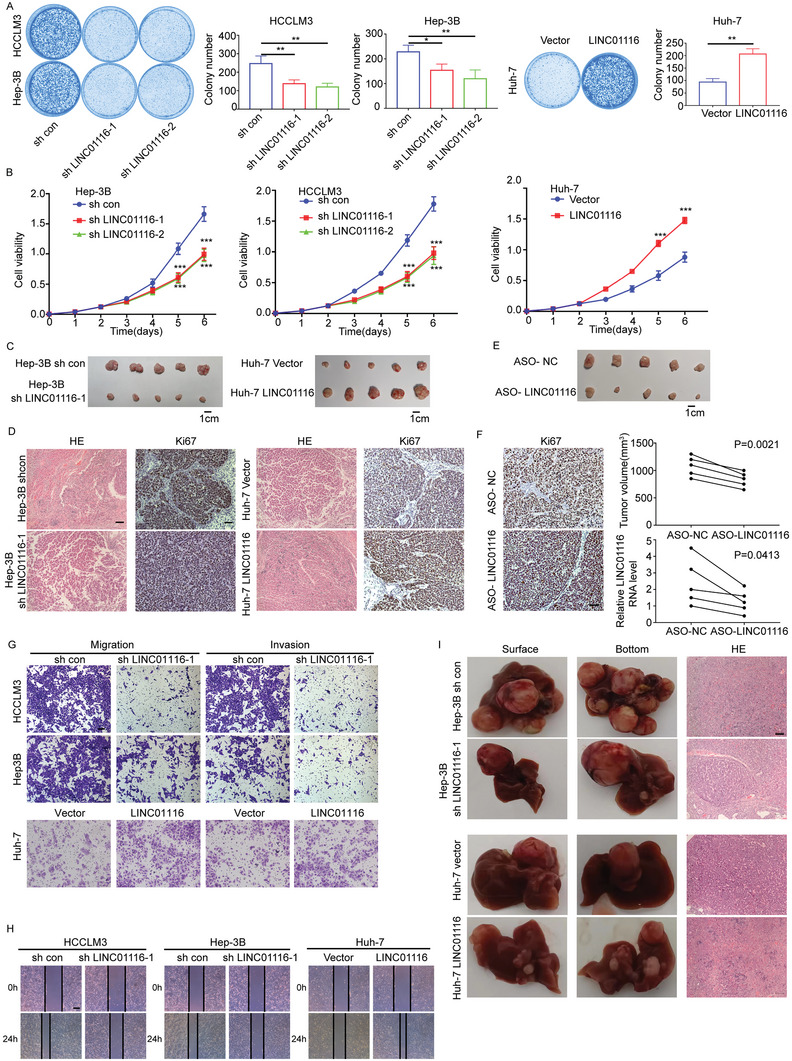
LINC01116 promotes HCC progression in vitro and in vivo. A) Representative images of colony formation assays and colony counts. B) CCK8 assays showed the proliferation ability of HCC cells. C) Representative pictures of the tumors from nude mice injected with the indicated cells (*n* = 5 mice/group). D) Representative images of hematoxylin and eosin staining (scale bars, 100 µm) and Ki67 staining (scale bars 50 µm) of subcutaneous xenograft model mice. E) Representative images of PDX tumors (*n* = 5 mice/group). F) Tumor Volume, expression of Ki67 (scale bars 50 µm), and relative expression of LINC01116 of two groups. G) Representative images of Transwell migration and Matrigel invasion assays for the indicated cells (scale bars 50 µm). H) Representative images of wound healing assays. Scale bar 400 µm. I) Representative images of specimens and hematoxylin and eosin staining of liver metastasis (scale bars, 100 µm). All experiments were performed three times, and the data are presented as the mean ± SD. ^*^ *p* < 0.05; ^**^ *p* < 0.01; ^***^ *p* < 0.001; ^****^ *p* < 0.0001.

Given that the aberrant expression of LINC01116 is associated with HCC progression and the survival of patients, we investigated the function of LINC01116 in vitro and in vivo. LINC01116 knockdown was performed in HCCLM3 and Hep‐3B cells, and overexpression experiments were performed in Huh‐7 cells. The efficiency of the shRNAs and lentivirus‐mediated overexpression were confirmed by qRT‐PCR assays, and we used these stable cell lines for downstream experiments (Figure S2A, Supporting Information). First, we used CCK8 and colony formation assays to explore the effect of LINC01116 on the proliferation of HCC cells. The results showed that LINC01116 knockdown resulted in decreased clone number, clone size, and proliferation ability. The LINC01116 overexpression group yielded the opposite results (**Figure** **2**A, B). Moreover, in vivo experiments showed that compared to the control group, the LINC01116 knockdown group exhibited significantly reduced tumor growth rate and volume. In contrast, we observed the opposite phenomenon in the LINC01116 overexpression group (Figure 2C; Figure S2B–E, Supporting Information). Furthermore, immunohistochemistry (IHC) of the excised tumor sections indicated that the expression of Ki67, a proliferation marker, in LINC01116 knockdown group was lower than that in the control group, while overexpression of LINC01116 produced the opposite effects (Figure 2D; Figure S2F, Supporting Information). To better evaluate the therapeutic role of intervening LINC01116, patient‐derived xenograft models were treated with antisense oligonucleotide drugs (ASO). The lower tumor volume and Ki67 expression in the ASO‐LINC01116 group compared with the ASO‐NC group indicated that pharmacological blockade of LINC01116 can limit tumor growth (Figure 2E,F; Figure S2G, Supporting Information). We then investigated the effect of LINC01116 on invasion and metastasis in HCC cells. We performed Transwell assays and wound healing assays. The results showed that the knockdown of LINC01116 in HCCLM3 and Hep‐3B cell lines reduced their motility and invasive ability, while LINC01116 overexpression had the opposite effect on these levels (Figure 2G,H; Figure S2H, Supporting Information). To explore the role of LINC01116 in HCC metastasis, we simulated intrahepatic metastasis by injecting tumor cells into the spleen. After 4 weeks, we found that the LINC01116 knockdown group exhibited reduced number and size of intrahepatic metastatic nodules compared with those in the control group, while the overexpression group showed opposite results (Figure 2I; Figure S2I,J, Supporting Information). These data suggest that LINC01116 promotes tumor progression.

### 2.3 LINC01116 Influences Tumor Progression by Reprogramming Lipid Metabolism

**Figure 3:**
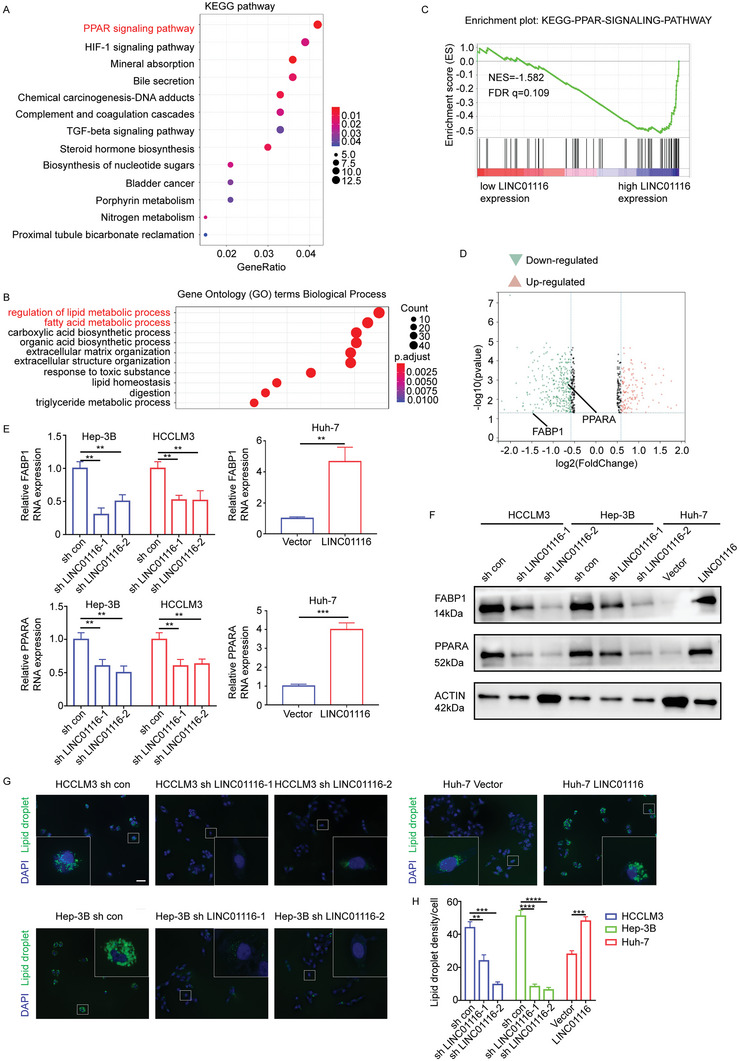
LINC01116 influences tumor progression by reprogramming lipid metabolism. A) KEGG enrichment analysis of LINC01116‐regulated genes. B) GO enrichment analysis for control and sh LINC01116 Hep‐3B cells. C) GSEA enrichment plot for PPAR pathway from Hep‐3B RNA expression. D) Volcano map of RNA sequencing data for control and sh LINC01116 Hep‐3B cells. E) qPCR assays showed the relative expression in the indicated cells. F) Western blot showed the expression of PPAR signaling pathway‐associated proteins. G) Immunofluorescence showed the density of lipid droplets in the indicated cells. White boxes showed representative pictures. H) Statistical analyses in Figure 3G. Scale bars 50 µm. ^**^ *p* < 0.01; ^***^ *p* < 0.001; ^****^ *p* < 0.0001, based on Student t test.

As the expression level of LINC01116 was closely related to the biological behavior of tumor cells, RNA‐seq was used to discover the altered genes after LINC01116 knockdown in Hep‐3B cells. Kyoto Encyclopedia of Genes and Genomes (KEGG) pathway analysis and Gene ontology (GO) analysis suggested that LINC01116 might be related to the PPAR signaling pathway and lipid metabolism (**Figure** **3**A,B). GSEA results also showed that these pathways were significantly enriched in the LINC01116 high‐expression group (Figure 3C). It is well known that the PPAR pathway is closely related to lipid metabolism. These findings suggested that LINC01116 is closely related to lipid metabolism. Among the enriched genes, FABP1 and PPARA, which are related to lipid metabolism and the PPAR pathway, were altered significantly (Figure 3D). To validate the results of the analysis, we performed validation using qRT‐PCR and western blotting (WB). The results showed that the mRNA and protein levels of PPARA and FABP1 were significantly decreased after LINC01116 knockdown (Figure 3E,F). Lipid droplets are generally considered to be cellular fatty acid pools combined with different lipid fluxes and control their use according to specific cellular requirements.^[^ ^21^ ^]^ To confirm the role of LINC01116 in lipid metabolic reprogramming, the density of intracellular lipid droplets was measured. Immunofluorescence (IF) showed that the density of intracellular lipid droplets decreased after LINC01116 knockdown, and the density of intracellular lipid droplets increased after LINC01116 overexpression (Figure 3G,H). These results suggest that LINC01116 may be involved in reprogramming lipid metabolism in cancer cells.

### 2.4 Competitive Binding to EWSR1 Between LINC01116 and RAD18 could Promote EWSR1 Stabilization

**Figure 4:**
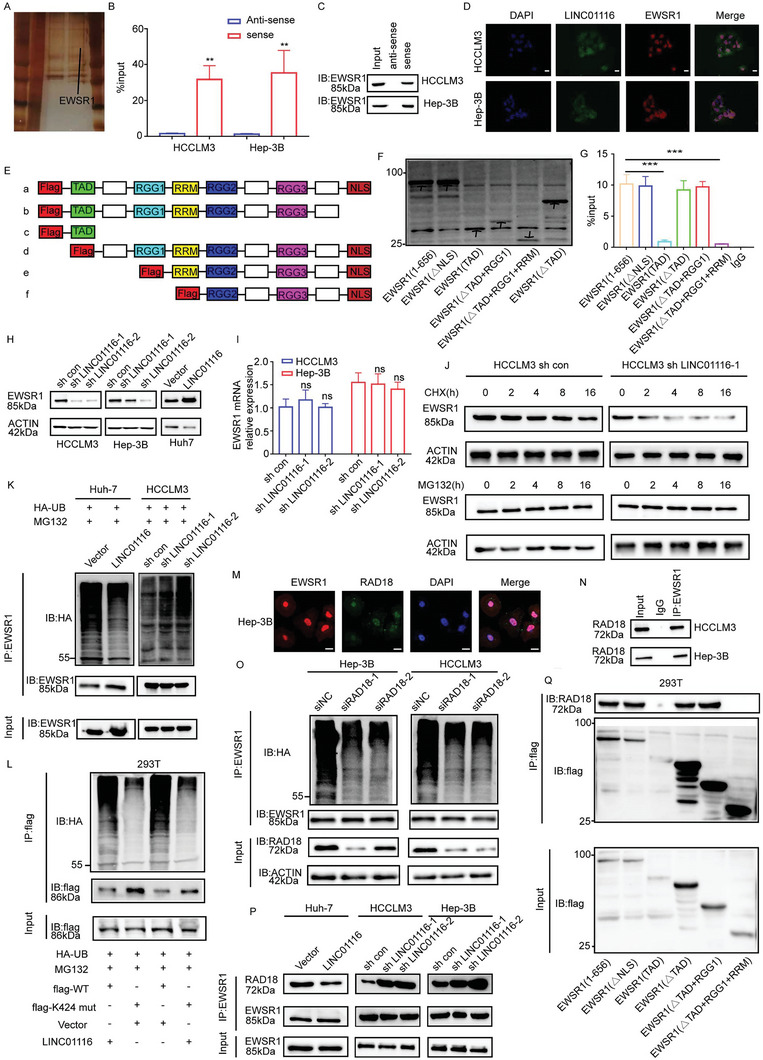
LINC01116 interacts with EWSR1 and stabilize EWSR1 protein. A) Silver staining showed different protein bands for mass spectrometry of the LINC01116‐protein complex pulled down by sense or anti‐sense in Hep‐3B cells. The arrow indicated the EWSR1 band. B,C) RNA pulldown and IB assays showed the interaction between EWSR1 and LINC01116. D) IF‐FISH assays showed the colocalization of LINC01116 (blue) and EWSR1 (red). Scale bars 20 µm. E) Schematic structures of EWSR1 proteins and five mutants were named as follows: a for EWSR1(1‐656), b for EWSR1(△NLS), c for EWSR1(TAD), d for EWSR1(△TAD), e for EWSR1(△TAD+RGG1) and f for EWSR1(△TAD+RGG1+RRM). F) WB assays showed the expression of EWSR1 and five mutants. The arrow points to each mutant. G) RIP and qRT‐PCR assays showed that the RRM fragment of EWSR1 is the LINC01116‐binding domain. H) WB showed the protein level of EWSR1 in HCC cells transfected with lentivirus compared to that of the control group. I) qRT‐PCR showed the EWSR1 mRNA relative expression in the indicated cells. J) Western blot assays showed the protein levels of EWSR1 in HCC cells treated with 20 µg/ml CHX or 25 µM MG132 for the indicated periods of time. K) Co‐IP assay showed the ubiquitination modification level of EWSR1 in the indicated cells. L) Co‐IP assay showed the ubiquitination modification level of EWSR1 in HCC cells co‐transfected with vector or LINC01116 and vectors expressing FLAG‐tagged WT or EWSR1 mutants (K424) as indicated treated with 25 µM MG132. M) Immunofluorescence showed that EWSR1 colocalized with RAD18. Scale bar, 20 µm. N) Co‐IP assays showed the interaction between EWSR1 and RAD18. O) Co‐IP assays showed alterations in EWSR1 ubiquitination levels in HCC cells transfected with si‐NC or si‐RAD18. P) Co‐IP assays showed the binding between EWSR1 and RAD18 protein in the indicated HCC cells. Q) Co‐IP showed the interaction between RAD18 and different EWSR1 domains in 293T cells transfected with different vectors. The data are presented as the mean± SD. ^**^ *p* < 0.01; ^***^ *p* < 0.001; ^****^ *p* < 0.0001, based on Student t test.

Many studies have shown that long noncoding RNAs in the nucleus regulate gene expression by binding to transcription factors, splicing factors, and other RNA‐binding proteins.^[^ ^22^ ^]^ To reveal the molecular mechanism of LINC01116‐mediated lipid metabolic reprogramming, we used RNA pulldown assays and mass spectrometry to identify important proteins that bind to LINC01116. Combined with the results of silver staining and mass spectrometry, we selected EWSR1, a target gene that may be related to lipid metabolism and the PPAR pathway,^[^ ^23^ ^]^ to clarify the mechanism by which LINC01116 affects lipid metabolism (**Figure** **4**A). Then, the interaction between LINC01116 and EWSR1 was verified using RNA immunoprecipitation (RIP)‐qRT‐PCR and RNA pulldown assays (Figure 4B,C; Figure S3A, Supporting Information). Additionally, we determined that LINC01116 colocalized with EWSR1 in the nucleus using IF‐FISH (Figure 4D). EWSR1 consists of four RNA recognition motifs (1 RRM and 3 RGGs). The RGG domain is an evolutionarily conserved sequence and at least 31 different protein isoforms contain the domain. It is important for inducing a stable G‐quadruplex and folding G‐quadruplex telomere DNA.^[^ ^24^ ^]^ The RRM (361–448 aa) is one of the most highly conserved nucleic‐acid‐binding domains in the C‐terminal region and is ubiquitously expressed in almost all cell types, which plays multiple roles in various cellular processes,^[^ ^25^ ^]^ such as m^6^A recognition.^[^ ^26^ ^]^ Therefore, to confirm which domain of EWSR1 binds to LINC01116, six FLAG‐tagged plasmids were constructed (Figure 4E,F). RIP‐qRT‐PCR results suggested that LINC01116 might bind to the RRM domain of EWSR1 (Figure 4G). These results indicated that LINC01116 interacted with EWSR1 in the nucleus.

EWSR1 plays an important role in gene expression, RNA processing, and cell signaling binding to a variety of transcription factors to form fusion proteins, which promotes the transcription of oncogenes and thus accelerates tumor progression.^[^ ^27^, ^28^, ^29^ ^]^ However, the role of EWSR1 in HCC has not been reported. Thus, we performed CCK8 assays and wound healing assays. We validated that EWSR1 could also promote HCC cell proliferation, and migration in vitro (Figure S3B–D, Supporting Information). To clarify the relationship between LINC01116 and EWSR1, we knocked down EWSR1 and LINC01116 respectively. The mRNA level of EWSR1 did not change after LINC01116 knockdown, while the protein level significantly decreased (Figure 4H,I). However, the expression of LINC01116 did not change after EWSR1 expression was altered (Figure S4A, Supporting Information). These results suggest that LINC01116 may affect levels of EWSR1 protein other than RNA.

We further showed that the knockdown of LINC01116 did not affect the protein synthesis, but may affect its degradation, suggesting that LINC01116 regulated EWSR1 protein stability through proteasome activity (Figure 4J). Consistent with the above results, overexpression of LINC01116 reduced EWSR1 ubiquitination. Knockdown of LINC01116 resulted in the opposite results (Figure 4K). We next identified the ubiquitination site of EWSR1 in HCCLM3 cells using LC‐MS/MS. Only one ubiquitinated lysine (K) residue (K424), which is located in the RRM domain of EWSR1, was confirmed (Figure S4B, Supporting Information). Moreover, we predicted the structure of EWSR1 through the UNIPROT website (https://www.uniprot.org) and visualized the K424 ubiquitination site (Figure S4C, Supporting Information). Then, we mutated the predicted site from lysine (K) to arginine (R) to confirm its role as a target for ubiquitination. Immunoprecipitation (IP) results showed that the K424 mutation significantly reduced EWSR1 ubiquitination compared with WT EWSR1, this mutation made the changes in EWSR1 ubiquitination caused by LINC01116 overexpression more pronounced, further confirming that K424 is the major ubiquitination site of EWSR1 (Figure 4L). These results prove that LINC01116 enhances the stability of EWSR1 protein by inhibiting ubiquitin/proteasome‐dependent degradation.

Seong B K A et al.^[^ ^30^ ^]^ showed that TRIM8 mediates the ubiquitination of EWSR1 in Ewing sarcoma. However, the role of E3 ubiquitin ligase on the ubiquitination of EWSR1 in HCC is unclear. Hence, we attempted to identify the E3 ubiquitin ligase involved in the proteasome‐mediated degradation of EWSR1 in HCC. We performed IP/mass spectrometry and identified that RAD18, a protein belonging to the RING family of E3 ubiquitin ligases that attach polyubiquitin to the target protein to promote their ubiquitination, could interact with EWSR1. To confirm whether RAD18 could modulate the ubiquitination of EWSR1, we executed immunofluorescence experiments and co‐IP assays (Figure 4M,N; Figure S4D, Supporting Information). The results showed that EWSR1 is bound to RAD18 in the nucleus. Moreover, we discovered that the protein level of EWSR1 was significantly elevated in RAD18 knockdown HCC cells, while its ubiquitination level was clearly decreased, suggesting that RAD18 acted as an E3 ubiquitin ligase that can degrade EWSR1 via the ubiquitin‐proteasome pathway in HCC cells (Figure 4O; Figure S4E, Supporting Information).

RNA pulldown and mass spectrometry analysis showed that LINC01116 did not bind to RAD18. However, we detected that the binding of EWSR1 to RAD18 was reduced after overexpression of LINC01116 and enhanced after knockdown of LINC01116, implying that LINC01116 may block the binding of EWSR1 to RAD18, which explains how LINC01116 protected EWSR1 from degradation (Figure 4P). To further prove that LINC01116 can block the binding between EWSR1 and RAD18, we performed co‐IP experiments and identified that RAD18 bound to the RRM domain of EWSR1, and interestingly, the RRM domain was also the site where LINC01116 interacted with EWSR1 (Figure 4Q). These data clarify that LINC01116 ultimately inhibits the ubiquitination of EWSR1 by competitively binding to the RRM domain of EWSR1 with RAD18. Overall, these data demonstrate that LINC01116 stabilizes EWSR1 protein by blocking RAD18‐mediated ubiquitination.

### 2.5 LINC01116 Plays a Cancer‐Promoting Role Through the EWSR1/PPARA/FABP1 Signaling Pathway

**Figure 5:**
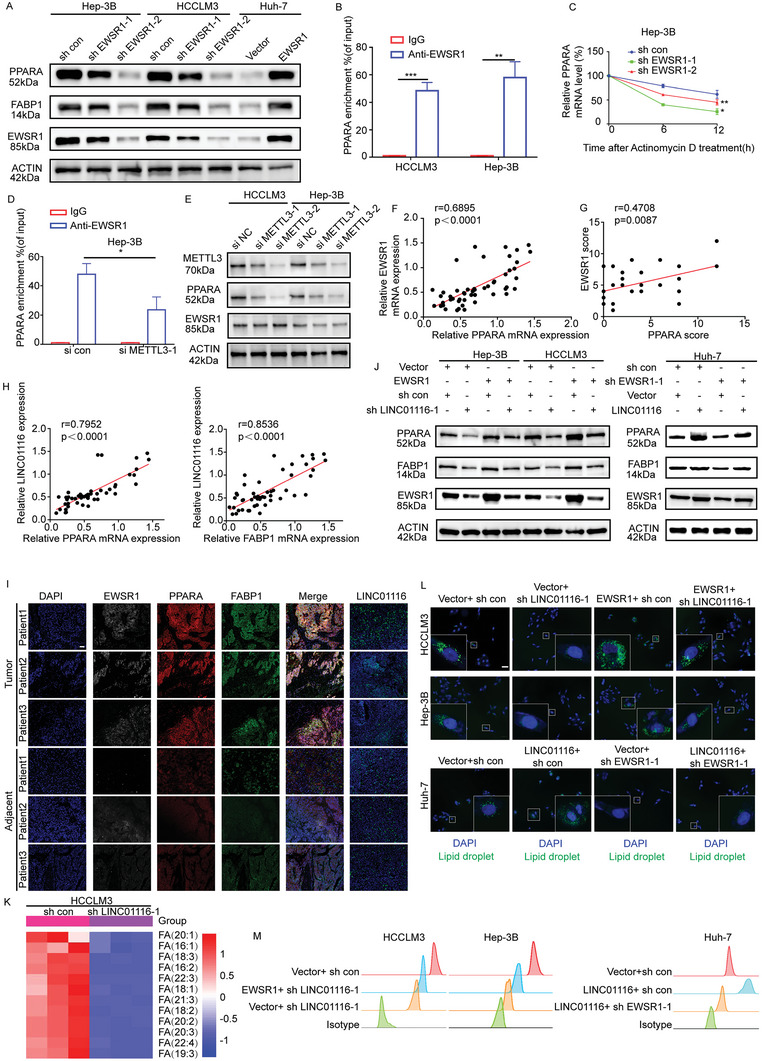
LINC01116 regulates PPARA and FABP1 expression via EWSR1 to shape lipid metabolism and promote cancer progression. A) WB assays showed the protein levels of PPARA, FABP1, and EWSR1 in the indicated HCC cell lines. B) RIP and qRT‐PCR showed the interaction between EWSR1 and PPARA mRNA in HCCLM3 and Hep‐3B cells. C. qRT‐PCR assays showed the mRNA levels of PPARA in Hep‐3B cell lines treated with actinomycin D (2 µg/ml) at the indicated time points. D) RIP‐qRT‐PCR showed the relative expression of PPARA mRNA in Hep‐3B cells transfected with si‐NC or si‐METTL3. E) WB showed the expression of METTL3, PPARA, and EWSR1 in the indicated cells. F,G) Pearson correlation analysis showed the correlation between PPARA and EWSR1. H) Pearson correlation analysis showed the correlation between LINC01116 and PPARA or FABP1. I) mIHC showed the distribution of PPARA, EWSR1, and FABP1 in HCC adjacent and tumor tissues. Scale bars 50 µm. J) WB showed the expression of PPARA, EWSR1, and FABP1 in the indicated cells. K) Heatmap showed lipidomic analyses of the indicated cells. L,M) Representative IF assay and flow cytometry results showed the level of neutral lipids stained with BODIPY 493/503 dye and DAPI in the indicated cells. White boxes showed representative pictures. Scale bars 50 µm. The data are presented as the mean± SD. ^*^ *p* < 0.05; ^**^ *p* < 0.01; ^***^ *p* < 0.001, based on Student t test.

Jiang W et al. revealed that EWSR1 may be related to the regulation of mRNA processing in HCC.^[^ ^31^ ^]^ Therefore, we hypothesized that the changes in PPARA and FABP1 expression induced by LINC01116 knockdown might be due to the effect of EWSR1 on their mRNA levels. First, we confirmed that the knockdown of EWSR1 in Hep‐3B and HCCLM3 cells resulted in decreased mRNA and protein levels of PPARA and FABP1. In contrast, overexpression of LINC01116 in Huh‐7 cells resulted in elevated mRNA and protein levels of PPARA and FABP1 (**Figure** **5**A; Figure S5A, Supporting Information). This suggested that EWSR1 may be involved in the RNA regulation of PPARA and FABP1, which is consistent with the findings of Jiang W et al. Recently, Xu X et al.^[^ ^26^ ^]^ identified that EWSR1 is a novel m^6^A reader that contains the RRM domain. It is known that m^6^A readers play an important role in mRNA transport and stabilization.^[^ ^32^ ^]^ Therefore, we hypothesized that EWSR1 may act as an m^6^A reader to regulate the expression of PPARA and FABP1. Next, we demonstrated that EWSR1 can bind to the mRNA of PPARA but not FABP1 by RIP‐qRT‐PCR (Figure 5B; Figure S5B,C, Supporting Information). Following treatment with actinomycin D, a transcription inhibitor for 0, 6, and 12 h, we found that the mRNA stability of PPARA deteriorated in the EWSR1 knockdown group, while the mRNA stability of FABP1 was unchanged (Figure 5C; Figure S5D, Supporting Information). To further confirm that EWSR1 regulated the mRNA stability of PPARA as an m^6^A reader, we performed RIP‐qRT‐PCR and found that the enrichment of PPARA mRNA was decreased in METTL3 knockdown Hep‐3B cells (Figure 5D; Figure S5E, Supporting Information). WB showed that the knockdown of METTL3 did not affect EWSR1 expression but PPARA protein expression (Figure 5E). Moreover, decreased expression of PPARA mRNA was not apparent compared with the control group when the global methylation inhibitor 3‐deazaadenosine (DAA) was introduced. Similarly, increased expression of PPARA mRNA was not obvious for the EWSR1 overexpression group with treatment (Figure S5F, Supporting Information). To further demonstrate the essential role of m^6^A in the regulation of PPARA, we predicted the m^6^A methylation sites by RMBaseV2.0 and designed a luciferase reporter inserting a wild‐type (WT) PPARA–3′UTR sequence or mutant (Mut) counterpart whose putative m^6^A sites were mutated (Figure S5G, Supporting Information). As expected, cells transfected with PPARA‐WT plasmid showed decreased luciferase activity when EWSR1 was silenced, whereas mutant cells showed no effect. And comparable results could be confirmed in EWSR1‐overexpressing group (Figure S5H, Supporting Information). In addition, in our own database, we found positive correlations between EWSR1 and PPARA by qRT‐PCR and immunohistochemistry (Figure 5F,G; Figure S5I, Supporting Information). Previous studies have shown that PPARA is a transcription factor for FABP1.^[^ ^33^ ^]^ Our findings combined with these results suggest that EWSR1 may alter the expression of FABP1 by regulating PPARA mRNA level in an m^6^A‐dependent manner.

Given the regulatory relationships of LINC01116 with EWSR1, PPARA, and FABP1 obtained above, we next analyzed their abundance in our own database. LINC01116 was positively correlated with EWSR1, PPARA, and FABP1 (Figure 5H,I). The same tendency was also noted for the nude mice experiments (Figure S5J, Supporting Information). To confirm regulatory relationships, rescue experiments indicated that knockdown or overexpression of EWSR1 could restore the expression changes of PPARA and FABP1 caused by LINC01116 alteration (Figure 5J). FABP1 is a FA‐binding protein involved in the transport of LCFAs.^[^ ^33^, ^34^ ^]^ Therefore, we hypothesized that LINC01116 might stabilize EWSR1 protein to upregulate PPARA/FABP1 expression which promoted lipid metabolic reprogramming and accelerated the uptake of LCFAs from the microenvironment. This was exemplified by the lipidomic analyses (Figure 5K). The uptake of lipids can remodel the cellular fatty acid pool.^[^ ^35^ ^]^ Therefore, cell lines were stained with the lipophilic dye BODIPY 493/503 to explore the intracellular lipid levels. Knockdown of EWSR1 reversed the increase in lipid droplets caused by LINC01116 overexpression. Overexpression of EWSR1 restored the reduction in lipid levels induced by LINC01116 knockdown (Figure 5L; Figure S5K, Supporting Information). Flow cytometry confirmed this conclusion (Figure 5M; Figure S5L, Supporting Information). In parallel, we determined the levels of metabolites containing phospholipids (PLs), triacylglycerols (TGs), lactate, and ATP in HCC cells. When the expression level of LINC01116 was downregulated, intracellular PL and TG levels were subsequently reduced. Intracellular PL and TG levels were increased after overexpression of LINC01116. Notably, the knockdown of EWSR1 attenuated TG and PL levels in LINC01116 overexpressing cell lines, while overexpression of EWSR1 increased TG and PL levels in LINC01116 knockdown cell lines (Figure S5M, Supporting Information). In addition, there was no significant correlation between lactate levels and LINC01116 expression levels (Figure S5M, Supporting Information). Intracellular ATP content positively correlated with LINC01116 expression levels, a trend that was abolished by the alteration of EWSR1 (Figure S5M, Supporting Information). ELISA results showed that compared with the control group, the culture supernatant of LINC01116 knockdown group contained more LCFAs, indicating that the ability of tumor cells to take up LCFAs was decreased after LINC01116 knockdown, and the uptake ability was restored to a certain extent after EWSR1 recovery (Figure S5N, Supporting Information).

Lipid metabolic reprogramming can provide tumor cells with molecules and energy to promote the biological behavior of tumors.^[^ ^36^, ^37^ ^]^ Considering that LINC01116 regulated lipid metabolism via EWSR1/PPARA/FABP1, we performed rescue experiments to explore the role of EWSR1 in LINC01116‐mediated tumor proliferation, migration and invasion. Rescue assays implied that downregulation of EWSR1 expression compensated for the increased proliferation, invasion, and migration abilities of HCC cells caused by exogenous overexpression of LINC01116. And the overexpression of EWSR1 enhanced the proliferation, migration, and invasion capacity of HCCLM3‐LINC01116‐sh1 and Hep‐3B‐LINC01116‐sh1 cells (Figure S5O,P, Supporting Information). The role of PPARA in LINC01116‐mediated lipid metabolic reprogramming, tumor proliferation, migration, and invasion was also examined by overexpressing or knocking down PPARA. WB confirmed the efficiency of knockdown and overexpression, as well as changes in downstream targets (Figure S5Q, Supporting Information). Rescue experiments showed that PPARA knockdown reduced the increase in lipid droplet, TG, phospholipid, ATP content, and LCFA uptake, as well as the increase in proliferation, invasion, and migration capacity caused by exogenous LINC01116 overexpression. In contrast, overexpression of PPARA showed the opposite tendency (Figure S5R–V, Supporting Information). These results suggest that LINC01116 activates the EWSR1/PPARA/FABP1 signaling pathway, thereby enhancing LCFA uptake and remodeling the cellular fatty acid pool to provide energy for proliferation and metastasis of tumor cells.

### 2.6 Tumor Cells Outcompete T Cells for Linoleic Acid

**Figure 6:**
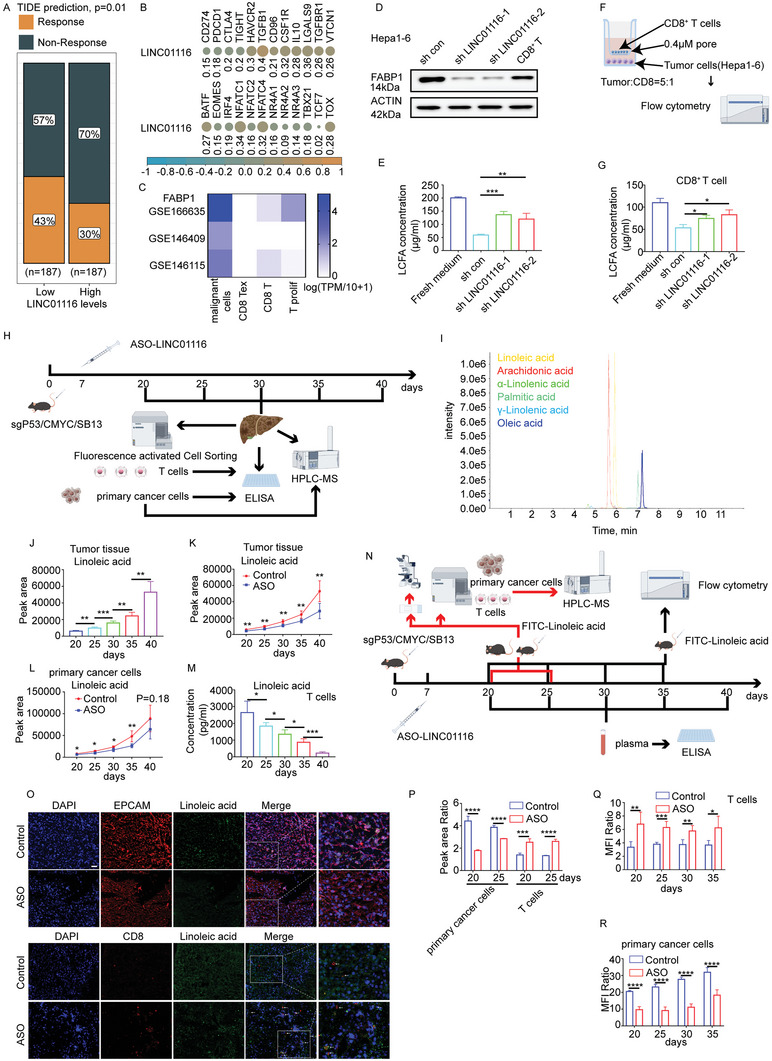
Tumor cells outcompete T cells for linoleic acid and impair T cell function. A) TIDE analysis showed that LINC01116 expression is related to the immunotherapy response. B) Correlation analysis between LINC01116 and immune checkpoints or transcription factors related to CD8^+^ T cell exhaustion. C) Expression of FABP1 mRNA in various types of cells in the TISCH database. D) WB assays showed the protein level of FABP1 in the indicated cells. E) The concentration of LCFAs in the culture supernatant was measured by ELISA. F) The schematic of co‐culture experiment. G. The concentration of LCFAs in the CD8^+^ T cells was measured by ELISA. H) Schematic of the experiment. I. Typical total ion chromatograms of fatty acids from tumor tissues. J) LC‐MS/MS analysis of linoleic acid in the tumor tissues at the different time points (*n* = 5/group). K) LC‐MS/MS analysis of linoleic acid in the tumor tissues from the control and ASO LINC01116 group at the different time points (*n* = 5/group). L) LC‐MS/MS analysis of linoleic acid in the primary cancer cells from the control and ASO LINC01116 group at the different time points (*n* = 5/group). M) The concentration of linoleic acid in the tissue‐specific T cells was measured by ELISA (*n* = 3/group). N) Schematic of the experiment for the competition for linoleic acid between hepatocellular carcinoma cells and T cells. O) The relative content of linoleic acid in tumor cells and T cells was demonstrated by immunofluorescence. Scale bars 50 µm. P) LC‐MS/MS analysis of linoleic acid in the tissue‐specific T cells and primary cancer cells from the control and ASO group (n = 3/group). Q) MFI Ratio represented the relative content of linoleic acid absorbed by the tissue‐specific T cells from the control and ASO group (n = 5/group). R) MFI Ratio represented the relative content of linoleic acid absorbed by the primary cancer cells from the control and ASO group (n = 5/group). All experiments were performed three times, and the data are presented as the mean± SD. ^*^ *p* < 0.05; ^**^ *p* < 0.01; ^***^ *p* < 0.001; ^****^ *p* < 0.0001, based on Student t test.

Initially, we identified LINC01116 as an RNA related to immunity and metabolism. We have revealed how LINC01116 affects lipid metabolic reprogramming, and next, we will explore its relationship with tumor immunity. TIDE analysis and single‐sample GSEA (ssGSEA) showed that patients with high LINC01116 and EWSR1 expression were not sensitive to immunotherapy and were positively correlated with CD8^+^ T cell dysfunction (**Figure** **6**A,B; Figure S6A–K, Supporting Information). Transcription factors culminating in CD8^+^ T cell exhaustion were also strongly correlated with LINC01116 and EWSR1 expression (Figure 6B; Figure S6K, Supporting Information). These results indicated that LINC01116 was related to T cell inactivation. The metabolic reprogramming of tumor cells not only contributes to their own growth but also influences the function of other cells in the microenvironment.^[^ ^38^ ^]^ Many studies have shown that tumor cells can outcompete T cells for large amounts of nutrients in the microenvironment through their transporters to maintain their energy supply, thereby impairing T cell survival and function.^[^ ^39^, ^40^ ^]^ The proliferation of T cells requires exogenous LCFAs for membrane synthesis, energy production, and TCR signaling.^[^ ^40^ ^]^ Considering that LINC01116 overexpression led to increased FABP1, an important protein for transporting LCFAs, we wondered whether high LINC01116 expression in tumors would deprive T cells of LCFAs uptake in the microenvironment, leading to T cell exhaustion and insensitive to immunotherapy. To confirm that the nutrient deprivation induced by LINC01116 led to T cell exhaustion, first, FABP1 protein expression in tumor cells was compared with that in CD8^+^ T cells. Tumor cells had a higher expression of FABP1 than CD8^+^ T cells in the TISCH database (Figure 6C; Figure S6L, Supporting Information). WB showed that the expression of FABP1 in CD8^+^ T cells was lower than that in Hepa1‐6 cells and the expression of FABP1 was downregulated in Hepa1‐6 cells after knocking down LINC01116 (Figure 6D; Figure S6M, Supporting Information). ELISA results showed that compared to the supernatants from the control group, the supernatants from the LINC01116 knockdown group had more LCFAs (Figure 6E). Then, we cultured spleen CD8^+^ T cells with Hepa1‐6 control and LINC01116 knockdown cells (Figure 6F). CD8^+^ T cells treated with Hepa1‐6 LINC01116 knockdown cells obtained more LCFAs (Figure 6G). Not unexpectedly, T cells co‐cultured with Hepa1‐6 control cells lost partial function, including proliferation, effector cytokine production, and cytotoxic molecule production, compared to those of LINC01116 knockdown cells (Figure S6N–U, Supporting Information). To determine which LCFAs were involved in the competition, targeted lipidomics approaches and ELISA for 6 typical LCFAs on MYC‐luc; sg‐p53 mice were performed at various time points^[^ ^41^ ^]^ (Figure 6H,I). Tumor tissues obtained more linoleic acid and palmitic acid than other lLCFAs accompanying tumor development (Figure 6J, Figure S7A,B, Supporting Information). The decrease in tumor uptake of linoleic acid was most pronounced after the administration of ASO LINC01116 drugs (Figure 6K, Figure S7C,D, Supporting Information). Identical results were obtained by targeted lipidomics approaches and ELISA on primary cancer cells (CD45^−^EPCAM^+^) which were isolated by fluorescence‐activated cell sorting (Figure 6L; Figure S7E–G, Supporting Information). In striking contrast, as the tumor progressed, the content of linoleic acid in tumor‐specific CD8^+^ T cells became increasingly scarce (Figure 6M). To further demonstrate the existence of competition for linoleic acid between tumor cells and T cells in vivo, we injected FITC‐Linoleic acid into MYC‐luc; sg‐p53 mice at different time points and performed a series of experiments (Figure 6N). Primary cancer cells acquired more linoleic acid than T cells, however, this trend reversed in ASO LINC01116 group (Figure 6O). Flow cytometry and LC‐MS/MS analysis showed that when we use ASO drugs, primary cancer cells absorb less linoleic acid. By marked contrast, in tissue‐specific CD8^+^ T cells, the capacity to transport linoleic acid obviously increased (Figure 6P–R; Figure S7H, Supporting Information). These results suggest that tumor cells outcompete T cells for linoleic acid.

### 2.7 Linoleic Acid Can Reverse T‐Cell Dysfunction Caused by LINC01116

**Figure 7:**
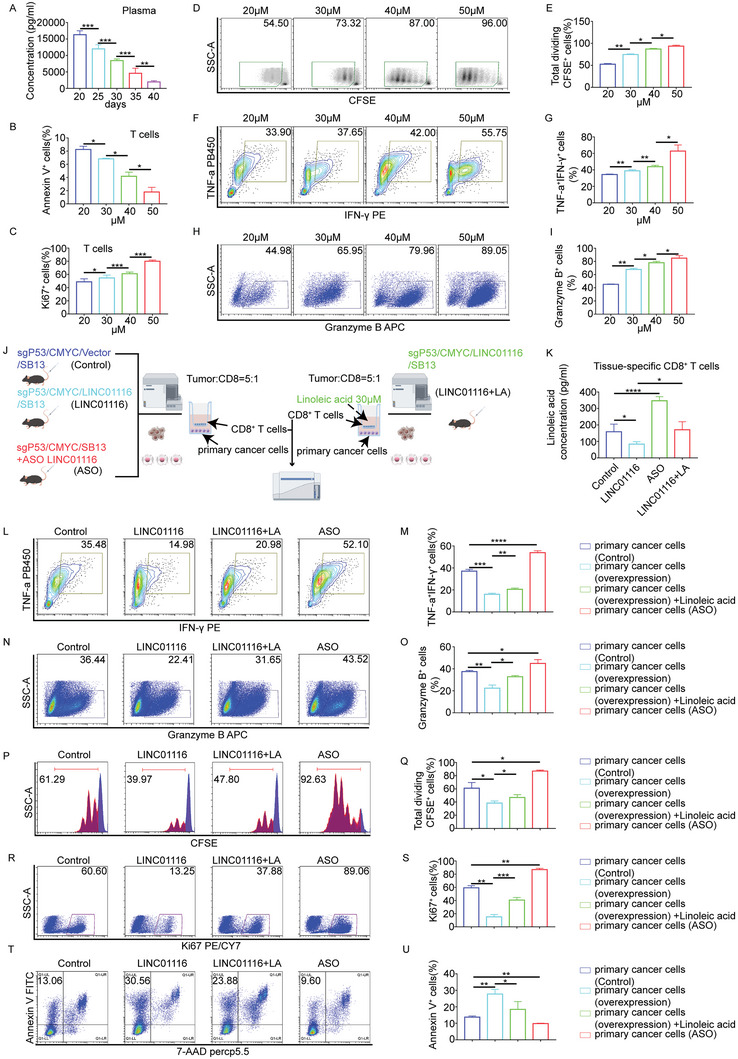
Linoleic acid can reverse T‐cell dysfunction caused by LINC01116. A) Plasma linoleic acid concentrations were measured by ELISA (*n* = 5/group). B) Statistical analysis of Annexin V^+^ cells cultured with linoleic acid (*n* = 3/group). C) Statistical analysis of Ki67^+^ cells cultured with linoleic acid (*n* = 3/group). D,E) Representative density plots (D) and quantification (E) of activated T cells cultured with different concentrations of linoleic acid (*n* = 3/group). F,G) Contour plots (F) and quantification (G) of TNF^+^IFN‐γ^+^ T cells cultured with different concentrations of linoleic acid (n = 3/group). H,I) Representative flow plots (H) and quantification (I) of Granzyme B^+^ T cells cultured with different concentrations of linoleic acid (*n* = 3/group). J) Schematic of effects of LINC01116 and linoleic acid on T cells. K) Linoleic acid concentration in tissue‐specific CD8^+^ T cells of the indicated group was measured by ELISA. L,M) Contour plots (L) and quantification (M) of TNF^+^IFN‐γ^+^ T cells for the indicated group (n = 3/group). N,O) Representative flow plots (N) and quantification (O) of Granzyme B^+^ T cells for the indicated group (*n* = 3/group). P,Q) Representative histograms (P) and quantification (Q) of activated T cells for the indicated group (n = 3/group). R,S) Representative flow plots (R) and quantification (S) of Ki67^+^ T cells for the indicated group (n = 3/group). T,U) Representative flow plots (T) and quantification (U) of Annexin V^+^ T cells for the indicated group (n = 3/group). ^*^p < 0.05; ^**^p < 0.01; ^***^p < 0.001; ^****^ *p* < 0.0001, based on Student t test.

We have shown that there is competition between tumor cells and T cells for linoleic acid. However, the effect of linoleic acid deficiency on T cell fate has not been clearly elucidated. First, we explored that plasma linoleic acid concentrations decreased as the tumor progressed (**Figure** **7**A). Then, considering the effect of lipotoxicity in vitro cell culture, we selected several corresponding plasma linoleic acid concentrations to culture T cells. Briefly, 50, 40, 30, and 20 µM corresponded to plasma linoleic acid concentrations at day 25, 30, 35, and 40. As expected, with the increase of linoleic acid concentration, T cells proliferated robustly, decreased apoptotic signaling, and got stronger cytotoxicity (Figure 7B‐I). To make the results more reliable, we performed rescue experiments using primary cancer cells (Figure 7J). First, we tested the expression of FABP1 in primary cancer cells and tissue‐specific CD8^+^ T cells (Figure S8A, Supporting Information). Figure S8A–C (Supporting Information) again confirmed the positive correlation between LINC01116 and EWSR1, PPARA and FABP1 expression and the role of LINC01116 in primary cancer cells. Tissue‐specific CD8^+^ T cells derived from the ASO group had a higher expression of FABP1 than those of the Control group (Figure S8A, Supporting Information). By contrast, tissue‐specific CD8^+^ T cells derived from the LINC01116 group showed lower expression of FABP1 (Figure S8A, Supporting Information). Moreover, following ASO treatment, tissue‐specific CD8^+^ T cells, and primary cancer cells showed similar FABP1 expression (Figure S8A, Supporting Information). Then, we measured the linoleic acid concentration in tissue‐specific CD8^+^ T cells by ELISA. Tissue‐specific CD8^+^ T cells derived from the LINC01116 group absorbed less linoleic acid than those of the Control group, while this trend was restored with linoleic acid supplementation (Figure 7K). The opposite results were observed in the ASO group (Figure 7K). Co‐cultured with primary cancer cells derived from the LINC01116 group, compared to the Control group, T cell proliferation and cytotoxicity were impaired, whereas apoptosis was increased. This trend could diminish when linoleic acid was added. Compared to the Control group, primary cancer cells derived from the ASO LINC01116 group improved T cell function (Figure 7L–U). These results show that LINC01116 controls T‐cell fate by regulating FABP1 expression and linoleic acid uptake.

### 2.8 Targeting LINC01116 Sensitizes HCC Cells to Immunotherapy

**Figure 8:**
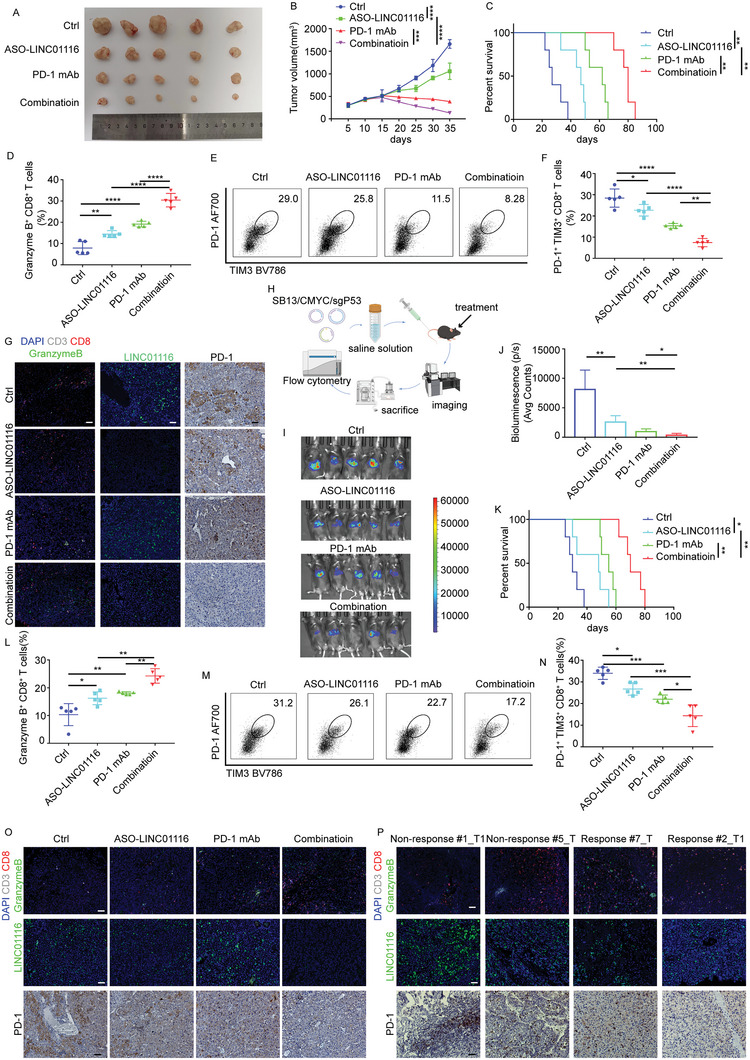
Targeting LINC01116 sensitizes HCC cells to immunotherapy. A) Representative images of subcutaneous HCC tumors from each group (*n* = 5 mice/group). B) Changes in tumor volume evaluated by tumor growth curves. Two‐way ANOVA. C) The Kaplan‐Meyer survival curves were compared between each group. Two‐sided Log‐rank test. D) The proportions of granzyme B^+^ T cells determined by flow cytometry. One‐way ANOVA. E,F) The proportions of PD‐1^+^TIM3^+^ T cells determined by flow cytometry. One‐way ANOVA. G) mIHC and IHC showed the distribution of granzyme B, PD‐1, and LINC01116 in each group. H) Schematic figure showing hydrodynamic tail vein injection. I) Representative images of bioluminescence pictures of the indicated tumor models. J) Bioluminescence imaging was used to monitor tumor growth. K) Survival curves of each group. L) The proportions of granzyme B^+^ T cells were determined by flow cytometry. One‐way ANOVA. M,N) The proportions of PD‐1^+^TIM3^+^ T cells were determined by flow cytometry. One‐way ANOVA. O,P) mIHC and IHC showed the distribution of granzyme B, PD‐1, and LINC01116 in each group. Scale bars 50 µm. p < 0.05 was considered statistically significant.

To assess whether targeting LINC01116 would enhance immunotherapy efficacy, we treated mice bearing subcutaneous Hepa1‐6 tumors with ASO LINC01116, anti‐PD1, or a combination of the two. Notably, in contrast to the anti‐PD1 group, the combination treatment showed a significant reduction in tumor growth, better treatment efficacy, and longer survival (**Figure** **8**A–C). Flow cytometry analysis showed that combination treatment markedly increased the population of granzyme B^+^ CD8^+^ T cells and decreased PD1^+^TIM3^+^ T cells (Figure 8D–F). Similar results were obtained by multiplex IHC (mIHC) staining (Figure 8G).

To further clarify the enhanced therapeutic efficacy of the combination treatment, we established an HCC animal model by hydrodynamic tail vein injection with SB13/c‐Myc/sgP53 and treated with ASO LINC01116, anti‐PD1 or the combination (Figure 8H). Consistent with the Hepa1‐6 tumor model, mice that received combination therapy, but not monotherapy, exhibited liver tumor regression and significantly prolonged survival (Figure 8I–K). ASO LINC01116 significantly enhanced granzyme B production in CD8^+^ T cells and decreased the population of exhausted T cells in HCC model mice. This was further augmented in mice that received combination treatment (Figure 8L–O; Figure S9A, Supporting Information). We also found that LINC01116 expression was higher in tumor tissues of patients who were not sensitive to anti‐PD1 treatment (Figure 8P). Meanwhile, we again confirmed that LINC01116 can activate EWSR1/PPARA/FABP1 signaling pathway by immunohistochemistry (Figure S9B, Supporting Information). Combined with previous studies, these data demonstrated that targeting LINC01116 could not only inhibit the proliferation of tumor cells but also sensitize tumors to immunotherapy. Therefore, LINC01116 may be a promising therapeutic target.

## 3 Discussion

Initially, noncoding RNAs were thought to be a group of functionless RNAs, but as research progressed, researchers discovered that they play an important role in regulating gene expression.^[^ ^42^ ^]^ In the field of tumor biology, there have been numerous studies on long noncoding RNAs in immunodeficiency models involving drug resistance, tumor proliferation, and metastasis. Recently, the role of long noncoding RNAs in immune cell function has received attention; however, the relationship between long noncoding RNAs and the immune microenvironment and immunotherapy of HCC still needs to be further studied.^[^ ^43^, ^44^ ^]^ In this study, LINC01116 was identified as a long noncoding RNA involved in the TME and metabolism using TCGA database. We characterized the roles of LINC01116 in HCC metabolism and the immune microenvironment. The novelty of our study is that we not only found the function of LINC01116 in tumor cells themselves but also explored its role in the microenvironment, clarifying the competition between tumor cells and T cells for linoleic acid (Figure S9C, Supporting Information).

Long noncoding RNAs located in the nucleus usually bind to RNA‐binding proteins and thus play a regulatory role in genes. We identified EWSR1 as a protein that binds to LINC01116. EWSR1 is involved in the proliferation, metastasis, and metabolic reprogramming of multiple tumor types.^[^ ^25^, ^45^ ^]^ It can bind to transcription factors to form fusion proteins and affect RNA stability and the alternative splicing process, thereby regulating the expression of downstream genes.^[^ ^46^, ^47^, ^48^ ^]^ However, prior to our work, the mechanistic connection between LINC01116 and EWSR1 and the biological function of EWSR1 in the TME and immunotherapy were undetermined. We verified that EWSR1 was also associated with HCC patient survival prognosis and promoted tumor proliferation and invasion. We also identified RAD18 as an E3 ubiquitin ligase that binds to EWSR1 to regulate its ubiquitination and demonstrated that LINC01116 stabilizes EWSR1 protein levels by blocking EWSR1 binding to RAD18. As a novel m^6^A reader, EWSR1 stabilizes PPARA mRNA, which in turn promotes FABP1 transcription by PPARA. Finally, the level of lipid intake is enhanced, and energy is provided for the biological function of tumor cells.

Multiple observational studies have found that cirrhosis from nonalcoholic fatty liver disease (NAFLD) is associated with an increased risk of HCC, and numerous reports have shown that the incidence of NAFLD‐related HCC is increasing yearly.^[^ ^49^ ^]^ In addition, to date, no drug has been approved for the treatment of nonalcoholic steatohepatitis (NASH). Recently, intestinal PPARα signaling was reported to promote NASH progression through FABP1 regulation of dietary FA intake, which suggests that FABP1 is a compelling therapeutic target for NASH treatment.^[^ ^34^ ^]^ This finding also suggests that the PPARA‐FABP1 signaling axis plays an important role in lipid metabolism. Our study demonstrated that LINC01116 can regulate the PARA‐FABP1 signaling axis and may be a novel target for NASH treatment.

Tumor cells, immune cells, and stroma constitute the TME. Metabolic reprogramming of tumor cells can lead to the consumption of key nutrients and the accumulation of waste products in the TME. These metabolic changes often affect the function of other cell types, especially immune cells, leading to a dysregulated immune response and decreased antitumor immunity. An increasing number of studies have shown that tumor cells can upregulate various transporters to competitively consume nutrients in the TME, thereby causing immune cell dysfunction.^[^ ^50^, ^51^ ^]^ Little is known about the competition between tumor cells and CD8^+^ T cells for LCFAs. In this study, we found that upregulation of FABP1 by LINC01116 accelerated the uptake of exogenous linoleic acid, resulting in the inactivation of T cells. Targeting LINC01116 increased tumor sensitivity to immunotherapy.

## 4 Experimental Section

### Patients and Tissue Samples

HCC and matched noncancerous border tissue samples were obtained during surgery at the First Affiliated Hospital of the University of Science and Technology of China. Tumors were classified according to the sixth edition of the TNM classification system. Ethical approval was obtained from the Research Ethics Committee of the First Affiliated Hospital of the University of Science and Technology of China, and written informed consent was obtained from each patient. The clinical characteristics of patients in this study are listed in Table S1 (Supporting Information).

### Transwell Assays

A total of 2 × 10^4^ cells were seeded in the upper layer of the Transwell membrane, while the lower chamber contained 10% fetal bovine serum to induce cell migration. After incubation at 37 °C in a 5% carbon dioxide atmosphere for 24 h, the upper layer of the membrane was wiped, methanol was used to fix the membrane for 10 min, and the cells passing through the membrane were stained with crystal violet for 20 min and observed under a microscope. For the invasion assays, chambers were covered with Matrigel (BD Biosciences, USA).

### Western Blotting

Collected cells were lysed using 1× RIPA buffer (Bestbio, BB‐3201) with protease inhibitor for 30 min on ice. The protein concentration was quantified by a bicinchoninic acid protein assay kit (Beyotime P0012S), and protein samples were mixed with loading buffer (Trans, DL101‐02) and heated at 95 °C for 10 min. After electrophoresis and transfer, the PVDF membrane was blocked with 5% w/v nonfat dry milk for 1 h and then incubated with antibodies overnight. The next day, the primary antibody was cleared, the bands were washed with PBST, and the secondary antibody was incubated for 1 h at room temperature and developed. Signals were detected using BeyoECL Plus (Beyotime, P0018S) and captured using a ChemiDoc Imaging System (Bio‐Rad). Antibodies were as follows: anti‐human EWSR1 (CST 11910 1:1000), anti‐human FABP1 (Proteintech 13626‐1‐AP 1:1000), anti‐human PPARA (Proteintech 15540‐1‐AP 1:1000), and anti‐human RAD18 (Proteintech 18333‐1‐AP 1:1000). Antibodies are shown in Table S2 (Supporting Information).

### CCK8 Assay

A total of 1000 cells were seeded in 96‐well plates and cultured with 90 µL medium and 10 µL CCK reagent for 1 h at 37 °C for each day of detection, and the absorbance at 450 nm was measured using a microplate reader.

### ELISA

ELISA was used to detect LCFAs, linoleic acid, TNF‐a, IFN‐γ and IL‐2 levels. In brief, cell supernatants were collected and centrifuged at 1000 × g for 30 min at 4 °C, and cell precipitates were discarded. Levels of LCFAs, TNF‐a, IFN‐γ and IL‐2 were measured by ELISA kits (Shanghai Fantaibio, Elabscience) according to the manufacturer's instructions. For linoleic acid, 10^5^ cells were washed by PBS and collected in a tube. 1 mL lysis buffer was used. Plasma was obtained by centrifuging clean blood for 15 min at 2 000 g at 4  °C. 10 µL plasma was used for ELISA. Levels of linoleic acid were measured by ELISA kits (Shanghai Yuanju Biotechnology Co., Ltd).

### RNA FISH

The FISH kit was purchased from Gemma Genes. In brief, 4% paraformaldehyde was used to fix cells, and then the slides were blocked and incubated overnight with a denatured probe. For the processing of tissue sections, dewaxing and proteinase K treatment were used. Finally, the slides were stained with DAPI and observed using fluorescence microscopy.

### Colony Formation Assay

A total of 1000 cells were seeded in 6‐cm Petri dishes and cultured with a complete medium for two weeks. The medium was discarded, fixed with methanol for 20 min, stained with crystal violet for 30 min, and washed three times with PBS. Colonies larger than 1 mm in diameter were recorded.

### Wound Healing Assay

Cells (8 × 10^5^) were seeded in 6‐well plates. The next day, a 200 µL pipette tip was used to make vertical and continuous scratches. Floating cells were washed off with PBS. Cells were cultured in a complete medium for 24 h and photographed at 0 h and 24 h using a microscope.

### Coimmunoprecipitation (co‐IP)

A total of 1 × 10^7^ cells were lysed using 1 mL of IP lysis buffer on ice for 30 min. After sonication and centrifugation, the supernatant was incubated with antibodies for 4 h and beads for another 2 h. After washing beads with wash buffer, protein samples were used for WB experiments. The antibodies were as follows: anti‐human EWSR1 (CST #11910 5 µg) and anti‐rabbit IgG (CST 3900 5 µg). Antibodies are shown in Table S2 (Supporting Information).

### Hydrodynamic Tail Vein Injection

PT3‐EF1a‐MYC‐IRES‐luciferase (MYC‐luc) and pX330 p53 (sgP53) were purchased from Addgene, and CMV‐sleeping Beauty 13 (SB13) was a gift from Professor Amaia Lujambio. Myc‐Luc (11.4 µg) or LINC01116‐Luc (11.4 µg), sgP53 (10 µg), and SB13 (5.35 µg) were dissolved in 2 ml of sterile 0.9% saline. Six‐week‐old male mice were used for the experiments. The solutions were rapidly injected into the mice via the tail vein within 4–8 s. The volume injected was ten percent of the body weight. Plasmids were prepared using an Endo Free Maxi Kit (Qiagen).

### T Cell Isolation and Co‐culture System

The spleen was homogenized, and single cells were suspended in 2 mL Red Blood Cell Lysis Buffer (Biolegend) for 1 min. The splenocytes were pelleted, and washed and CD8^+^ T cells were selected through magnetic sorting with a CD8a^+^ T cell isolation kit (Miltenyi). Single‐cell suspensions were obtained by treating tumor 0tissues with a Multani Biotec kit. Endogenous tumor‐specific T cells were gated on CD45^+^CD3^+^CD8^+^ cells. CD8a^+^ T cells were resuspended at 2 × 10^6^ cells per ml in RPMI culture medium containing 5 µL CD3/28 beads (Thermo), 5 µg mL mouse recombinant IL‐2, and 40 µM 2‐mercaptoethanol. T cells were incubated at 37 °C for 4 days. To set up the co‐culture system, primary cancer cells or Hepa1‐6 cells were seeded overnight. T cells were then added to the culture. For CFSE, the cell trace CFSE Cell Proliferation Kit (Thermo, C34570) was used following the protocol. For cytokine staining, lymphocytes were incubated in a culture medium containing PMA (5 ng mL^−1^), ionomycin (500 ng mL^−1^), Brefeldin A (1:1000), and Monensin (1:1000) at 37 °C for 4 h. All cells were collected and analyzed by flow cytometry (FACS).

### Flow Cytometry Analysis

Single‐cell suspensions were obtained by treating tissues with a Multani Biotec kit. After treatment with red blood cell lysis buffer, the suspensions were blocked with CD16/32 for 10 min and incubated on ice for 30 min with the corresponding antibodies. After fixation and permeabilization, the suspensions were incubated on ice in the dark for staining antibodies and analyzed using a Cytoflex instrument. Antibodies are shown in Table S2 (Supporting Information).

### Immunofluorescence (IF)

Cells were fixed with 4% paraformaldehyde for 20 min and then treated with 0.2% Triton X‐100 in PBS for 10 min. Cells were blocked with 5% BSA for 30 min and then incubated with primary antibody overnight at 4 °C. Cells were incubated with secondary antibody for 30 min the next day. Cells were incubated with DAPI and analyzed by laser confocal microscopy (Zeiss, Germany). The antibodies were as follows: anti‐human EWSR1 (Abcam ab133288 1:200) and anti‐human RAD18 (Proteintech 18333‐1‐AP 1:100). Antibodies are shown in Table S2 (Supporting Information).

### LC‐MS/MS Analysis

The sample stored at −80 °C refrigerator was thawed on ice. A 200 µL methanol/acetonitrile (1:1, v/v) solution containing internal standard was added to the 20 mg sample. The mixture was homogenized by a grinder (30 HZ) for 20 s The protein was precipitated at a low temperature (−20 °C) for 30 min. The sample was centrifuged at 12 000 rpm for 10 min (4 °C). All supernatant was collected and transferred. Repeat the extraction once and combine the supernatants. The eicosanoids in supernatants were extracted using PolySery MAX SPE columns (ANPEL). Prior to analysis, the eluent was dried under a vacuum and redissolved in 100 µL of methanol/water (1:1, v/v) for UPLC/MS/MS analysis. For data analyses, an ExionLC UHPLC System combined with an AB SCIEX TripleTOF 5600+ System was used, and data acquisition was carried out with Analyst1.7.1 software.

For each cell sample, 200 µL water was added to the sample. After 30 s vortex, the samples were frozen and thawed with liquid nitrogen 3 times. Then the samples were sonicated for 10 min in an ice‐water bath. Add 480 µL MTBE: MEOH = 5:1 (containing internal standard) to the EP tube. After 30 s vortex, then the samples were sonicated for 10 min in an ice‐water bath. Then the samples were incubated at −40 °C for 1 h and centrifuged at 3000 rpm (RCF = 900(×g), R = 8.6 cm) for 15 min at 4 °C. 300 µL of supernatant was transferred to a fresh tube and dried in a vacuum concentrator at 37 °C. Then, the dried samples were reconstituted in 50 µL of 50% methanol in dichloromethane by sonication on ice for 10 min. The constitution was then centrifuged at 12 000 rpm (RCF = 16 200(×g), R = 8.6 cm) for 15 min at 4 °C, and the supernatant was transferred to a fresh glass vial for LC/MS analysis. LC‐MS/MS analyses were performed using an UHPLC system (Vanquish, Thermo Fisher Scientific), equipped with a Kinetex C18 column (2.1 mm × 100 mm, 1.7 µm, Phenomen). The mobile phase A consisted of 40% water, 60% acetonitrile, and 10 mmol L^−1^ ammonium formate. The mobile phase B consisted of 10% acetonitrile and 90% isopropanol, which was added with 50 mL 10 mmol L^−1^ ammonium formate for every 1000 mL mixed solvent. The analysis was carried out with elution gradient as follows: 0–1.0 min, 40% B; 1.0–12.0 min, 40%–100% B; 12.0–13.5 min, 100% B; 13.5–13.7 min, 100% ∼ 40% B; 13.7–18.0 min, 40% B. The column temperature was 55 °C. The auto‐sampler temperature was 4 °C, and the injection volume was 2 µL (pos) or 2 µL (neg), respectively.

### Quantification of Neutral Lipids, TGs, PLs, Lactate and ATP

The levels of TGs (Solarbio Life Science), PLs (Solarbio Life Science), lactate (Beyotime), and ATP (Beyotime) were measured by kits. Experiments were performed according to the manual provided by the institution. After fixation with 4% paraformaldehyde and permeabilization with 0.2% Triton X‐100, the cells were stained with 2 µM BODIPY 493/503 for 40 min in darkness. After washing with PBS three times, the cells were treated with DAPI and observed by laser confocal microscopy (Zeiss, Germany).

### RNA Immunoprecipitation (RIP)

A total of 1 × 10^7^ cells were washed twice with DPBS and lysed with 1 mL RIP buffer (containing cocktail and ribonuclease inhibitor) (Thermo) for 30 min on ice. After sonication and centrifugation, the supernatant was incubated with the corresponding antibody for 4 h and then incubated with beads that had been washed and blocked for 2 h. Finally, the beads were eluted for WB and qRT‐PCR experiments. The antibodies were as follows: anti‐human EWSR1 (CST 11910 5 µg) and anti‐rabbit IgG (CST 3900 5 µg). Antibodies are shown in Table S2 (Supporting Information).

### Cell Lines

Huh‐7, HCCLM3, Hep‐3B, and Hep‐G2 cell lines which were purchased from the American Type Culture Collection (ATCC, USA) were authenticated by STR profiling. MHCC97H, and MHCC97L cell lines were purchased from the Shanghai Cell Bank of the Chinese Academy of Sciences.

### Animal Experiments

All animal experiments were approved by the Ethics Committee of the University of Science and Technology of China. Nude mice were housed in a specific pathogen‐free (SPF) animal room. Six‐week‐old male mice were used for all animal models. For the subcutaneous tumor model, 1 × 10^6^ cells were injected subcutaneously into nude mice. The formula A × b^2^ × 0.52 was used to measure the tumor volume using calipers, where a and b represent the tumor length and width, respectively. For in vivo liver metastasis, 1 × 10^6^ cells were injected through the spleen into the body. For the MYC/sg P53/SB13 model treatment, 10 nmol ASO‐LINC01116 and ASO‐NC (RIB‐BIO) were intraperitoneally administered once every three days beginning on day 7 post‐injection of plasmids. For PDX model, five specimens were obtained. Then, tumor tissue from the same patient was divided into two equally sized tissue blocks and inoculated subcutaneously into 2 NSG mice. One mouse was treated with ASO NC and the other with ASO LINC01116. For subcutaneous tumor treatment, 10 nmol ASO LINC01116 and ASO NC were intratumorally administered once every three days beginning on day 7 after tumor inoculation. For anti‐PD1 treatment, 100 µg anti‐PD1 (InVivoMAb, BE0146) was intraperitoneally administered once every three days beginning on day 7 after plasmid injection or tumor inoculation. Mice were randomized into groups with similar tumor volumes prior to the initiation of treatment. For linoleic competition experiments, mice were injected i.p. with 50 µg FITC‐linoleic (Qiyue biology, Xi An, China) in 50 µL DMSO, 90 min, before sacrifice and cell isolation.

### Immunohistochemistry

After paraffin embedding, sectioning, deparaffinization and antigen retrieval (Vector Labs, USA), formalin‐fixed tissues were incubated with primary antibodies and secondary antibodies and further incubated with ABC solution (Vector Labs, USA) and DAB solution. Hematoxylin (Sigma, USA) was used to stain the nuclei. Antibodies are shown in Table S2 (Supporting Information).

### RNA Pull‑Down Assays

A total of 1 × 10^7^ cells were washed twice with DPBS and lysed with 1 mL RIPA buffer (containing cocktail and ribonuclease inhibitor) (Thermo Fisher Scientific) for 30 min on ice. After sonication and centrifugation, the supernatant was incubated with the corresponding probes for 4 h and then incubated with streptavidin‐conjugated agarose magnetic beads (Thermo Fisher Scientific), which had been washed and blocked for 2 h. Finally, the beads were eluted for WB and qRT‐PCR experiments. DNA probes are shown in Table S3 (Supporting Information).

### Lentivirus and siRNA Transfection

Full‐length LINC01116 was inserted into the pCDH‐CMV‐MCS‐EF1‐Puro vector, and sh‐LINC01116 plasmids were inserted into the pLKO.1 vector. Empty plasmids were used as controls. Sh‐EWSR1, si‐METTL3, and si‐RAD18 were purchased from Tongyong (Anhui, China). Flag‐tagged and HA‐tagged expression vectors and mutants were provided by Tongyong (Anhui, China). For the transfection of siRNAs and plasmids, cells were transfected using PEI according to the manufacturer's instructions. Target sequences are shown in Table S3 (Supporting Information).

### Real‐Time PCR

Total RNA was extracted using the GeneJET RNA Purification Kit (Thermo, K0732) according to the manufacturer's instructions. Five hundred nanograms of total RNA were used for cDNA synthesis using PrimeScript RT Master Mix (Perfect Real Time) (Takara, RR036A). RNA levels were measured with gene‐specific primers using TB Green Premix Ex Taq II (Tli RNaseH Plus) (Thermo, RR820A). The results were normalized to ACTB. Target gene primers are shown in Table S3 (Supporting Information).

### Methods for Obtaining Public Datasets and Gene Sets and Bioinformatics Analysis

Gene expression data and clinical survival data were downloaded from TCGA GDC data portal (https://portal.gdc.cancer.gov/). Pancancer survival information was downloaded from the University of California Santa Cruz (UCSC) Xena database (https://pancanatlas.xenahubs.net). Progression‐free survival time and survival status data from liver cancer patients were extracted to analyze the progression‐free survival difference after grouping according to LINC01116 expression. TIDE (http://tide.dfci.harvard.edu/login/) analysis based on a gene expression matrix was used to evaluate the possibility of tumor immune escape in tumor samples and the therapeutic effect after immunotherapy. Thorsson et al.^[^ ^52^ ^]^ conducted a comprehensive analysis and report on the immune map of cancer in 2018, and 24 immune checkpoint inhibitory molecules for the analysis of this study were extracted. Zheng et al.^[^ ^53^ ^]^ analyzed and reported a gene set composed of 82 genes that could reflect and quantify T‐cell depletion in liver cancer by single‐cell sequencing. This gene set and defined it as CD8_TEX_HCC and further analyzed and processed it by ssGSEA extracted.^[^ ^53^ ^]^ R software (version 4.1.0) and the corresponding R packages were used for subsequent bioinformatics analysis and corresponding graphical visualization.

### Statistical Analyses

Statistical analysis was performed in GraphPad Prism (version 7, San Diego, CA, USA). Data were expressed as the mean ± standard deviation (SD) and were compared by Student's t‐test (two groups) or one‐way ANOVA (more than two groups). The tumor growth results were analyzed by using a two‐way analysis of variance (ANOVA). Kaplan–Meier survival curves were compared using the log‐rank test. A *p*‐value of less than 0.05 was considered statistically significant.

## Conflict of Interest

The authors declare no conflict of interest.

## Author Contributions

K.M., J.C., and Y.L. contributed equally to this work. K.M., J.W., Q.H., Y.L., L.L. performed conceptualization; K.M., J.C. performed Data curation; K.M., X.G., and S.L. performed investigation; K.M. performed visualization; K.M., J.C., Y.L., C.J., and N.Z performed methodology; K.M. wrote the original draft; Y.L. performed the formal analysis; L.S., S.Z, and X.L. provided resources; T.C. worked with software; Y.L., L.L. performed supervision; Y.L. and L.L. funded acquisition; Y.L. and L.L. performed validation; Project administration, L.L.; Y.L. and L.L. wrote, reviewed and edited the original draft; All authors read and approved the final manuscript.

## Supporting information

Supporting Information

Supporting Information

Supporting Information

Supporting Information

Supporting Information

Supporting Information

## Data Availability

The data that support the findings of this study are available from the corresponding author upon reasonable request.
