# Supplementary material for: Hepatocellular Carcinoma LINC01116 Outcompetes T Cells for Linoleic Acid and Accelerates Tumor Progression
Source: Adv Sci (Weinh). 2024 Mar 9;11(21):2400676. doi: 10.1002/advs.202400676 (PMC11151013; doi:10.1002/advs.202400676)
Supplement: Supplementary file 1 — Supporting Information [file ADVS-11-2400676-s001.pdf]

ADVANCED SCIENCE
Open Access

Supporting Information

for *Adv. Sci.*, DOI 10.1002/advs.202400676

Hepatocellular Carcinoma LINC01116 Outcompetes T Cells for Linoleic Acid and Accelerates Tumor Progression

Kun Ma, Junhui Chu, Yufeng Liu, Linmao Sun, Shuo Zhou, Xianying Li, Changyong Ji, Ning Zhang, Xinyu Guo, Shuhang Liang, Tianming Cui, Qingsong Hu, Jiabei Wang*, Yao Liu* and Lianxin Liu**

Fig. S1

A

lncRNA	Patients' survival information from the UALCAN database(P value)	Patients' relapse free survival information from the LnCAR database(P value)
AP000695.2	0.0027	No significant differences were observed between two groups
LINC01511	0.003	No significant differences were observed between two groups
LINC01116	0.013	0.0038
AC016405.2	0.024	No significant differences were observed between two groups
MCM3AP-AS1	0.03	0.25

B

>NR_040001.2 Homo sapiens long intergenic non-protein coding RNA 1116 (LINC01116), long non-coding RNA
CAGGAAATGACCCGAACTGCCAGCCTGCGCCTTTGCAGCCGGCCCTCGCTTTGCTGAAGACGAGCAGCTCCCACCAAAGTCTTGCCTCCCCTTACCCCGAAA
GCCCCGCTCAGTTCAATATTTCAAGTGAAAATCTGCCTGTTCGAAAAGAAAATATTCACAAATGAGCAGTGTATTAGAAGACAACTGAATTGCTTCTAAGAATGGG
TCTCACTCTGCCATCACCCAGGCTGGAGTGTGGTGGCACCATCATGGCTCACTGCAGCCTTGAACTCCTGAGCTCAAGTCATCCTCCCACCTCAGCCTTCCGA
GTAGCTGGAACTGCAGGTGTGAGCCACCATGCCTGGCTGATTTGTCTTTTTAAATTTTTTATAGAGACCGAGTCTCAACTATATTGTCCAGGCTGGAAAAGAACTT
CTTTCCCTCCAAGTGATAACCTTTTCCAGACAAGTCAGCTTAAAAACTGACCCAAAGGCCCTGAAGTACACAGTTTTCTTGGAGAAAGAAAAAAATAAGTGAAAA
GGACTTGCTGACTTGCTAATTCATTTTGGGGCCTGTGGGTAACATCAGAATGGCAAAGCACTTGGGGCTTTTTTCTAATTTGTATTTTTTTAATCTTCTGAGAAATG
ACTTTTATATGCCAAGATTCATTCCATGATTAGTTTTTATGACCAAGTTATGAACGCTTTTGAATATGGGGACCTAGAATAGAAATGCTAACCTACCTGCAAGGAGAG
CTCTGTGATTGGCATCCCCATCATGCTGTAACTGACACAAAATAAACTTAGGGTAATTGCCGCATAGTGTAACTTTAATGTGTGAGCATACTGTAACATCTGACAAG
CTTCTTGATCTAGATCAGCAATGTGTAACCCATTCATTGTTGTCACTCTCAACAAGGCCTTATGGTCAAACATCATCACTCTCACTAAAGAGATAGTTTACTGAAATA
TACTGAGATTTTGAAACTGGAGTCAAGAGGTAGCACACTGTTTTATGTTGATCCATTTAATGGTCCTTGACAGCCAATAAAAAGACACTGGAAGAATACGTGAATG

>NONMMUT038000 MOUSE LINC01116 DNA sequence:
CTGATGGGCAGGAAAAGGCTGGAACGAAAAGCCCTGGCCTTTGCTGGCCTCGATGGCTTTGCAGAAGAGAGGTGGCCTCCACCCACGTCTCCCCTCCCCCTC
CGCGCCACACAGCAGTACTGCAGTTTGTCACCTGCTTGGCTGGCCCCCTTAACAGTTGAAGCTCTGTTCCTCCTATGAAAGACTCAGCTACTCTATCTCCTGGC
TGAAGCCTCCAAGCTCCCCGAACACTGTGGTCCCTGAGGCTGTGAGACTCTCTCTTGCAAGTCAGCACCTGAGGATCCCTGGTCCAAGCTTCATTCCTCTGGG
TCCAAGAACCTAGAAGAAAAGCCTGGAGCCACACACTCACCTGGGTGAAGCCATAAGGAGGTCTTTTCAAGGCCTGACTAAACACCTAAGAGAAGCAACTTAAG
GAAGAAAGGACTTAGTTGGATAACGGCTCAGAGAGTCCACCACATCAGGGAAGCCTGGTAGCTGGAGTGAGTCAGACCACTGTGGCTGAAGCTTCCCACATGA
TGTGGACCACAAGCAAACAAACAAGAACAAGGAAATCTGGCCTGTCACTCTCAATGACTATCACCAGTGACCCTCCAGTGCCAGCCATGCTTCCTACTACAAAG
GCTCCACAACCTCCCAAAACAGGGGACCAAGGGTTCACACACATGTGCCTATAGAGGACACATCACAGTCAACATAAGAGTGGAACTGAGACACAGAGTGGAA
GCCAGAGGGAAGAACACTGATCCTACATCTTAATAAATGGCCATAGAGGGACTGCCACTTGCTATTAAGTAGCATGTAAACCTCAGAGACTGTCTGTCATCTTGTG
CTTTAAAGGCTACTTTAAGTGACAATGTTAATGATGTATCTAGTGATGACAGTTGGCGAAACAAAGCCCAGCCATCAGAAGCTTATTGAATATAAGTGTCTAGCACA
CCGCGAGCCACCCAGCAATCTAATGTGGAAATGCAGATGTGCCACCAAAGACACAGACAGTTCATGACCTCACATGGTGTCTCTGCAGGACACTCACCTGTGT
GGATATGACAGAGCATGGGTCATTTGGACTGCCGAAGGGTGTTGTTTTCCTGGGGAGATGGCTCAACCAGGAAAATGTTGGCAGCCTAAGCCTGAGGACCT
GAATTAAGACCCCAGAGCCCATGTTACAGGGAGTCTGGAGTGACAGTGCATACTTGGCGTGCTGGTCCTGAGGAGTTGGAGTCAAAGGCATGCCCAGGGCT
CCAGAGCCAGTCAGTTCTGTGAGCTCAAGCTCAAGGAGAGACCTTGTCATAGAAAACAAGATGGCGGCTGCTTAGGAAGGACGTTTGAGCTGATCTCTGTAC
TTCACTCACACATGCACACACACACAGGTACACATTTTATTTTAGAACCCATGTTCATAAAGGAGAGTAGAGATGGGTAAGTATGAAGACAGCACAGAAAATAGTT
GAAGCTTTGATGCAATTATTCAGCAGCTCCGACCTTTGATGGTTACACCTAGACTTGTGATTTGACTTAAGTGGTTTTCTTTTTTATGGAGAAAAGAGTTTTTGAC
TTCCTATACAGTACCAGGTAAATTATGCCTTAGATAAAATAATCTGTGTATGGAATGGTTGTAACTAACTATAGATCACTGTAAATCCACCAAGCACATCTGAGGTAG
GGCAGGCTAAGCAGTGGTATTCGGAAGGTTGGACAGGTTATTAGGCTTCAGTTGGTCTATTGGTGTGGAATCCTATCACGAGTTAAAGTTTCTTTAAGAGTATAG
CTAGTGTAACTCTTACACTCTGCACTGAGAATTAGTACAGAAAGGGGCT

C

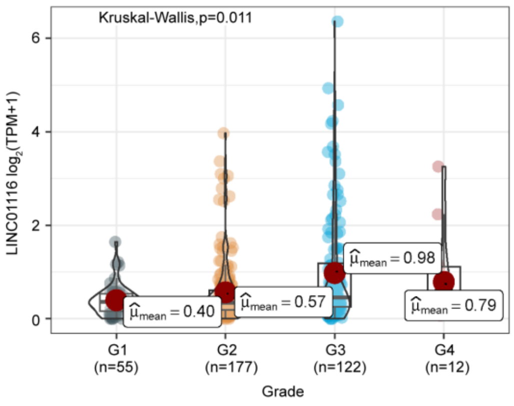

Figure S1 LINC01116 is associated with a high degree of malignancy hepatocellular carcinoma

A. Correlation between lncRNA and patient survival. **B.** The sequence of LINC01116 of Homo sapiens and Mus musculus. **C.** Box plot of LINC01116 expression versus tumor grade of HCC. $p < 0.05$ was considered statistically significant.

Fig. S2

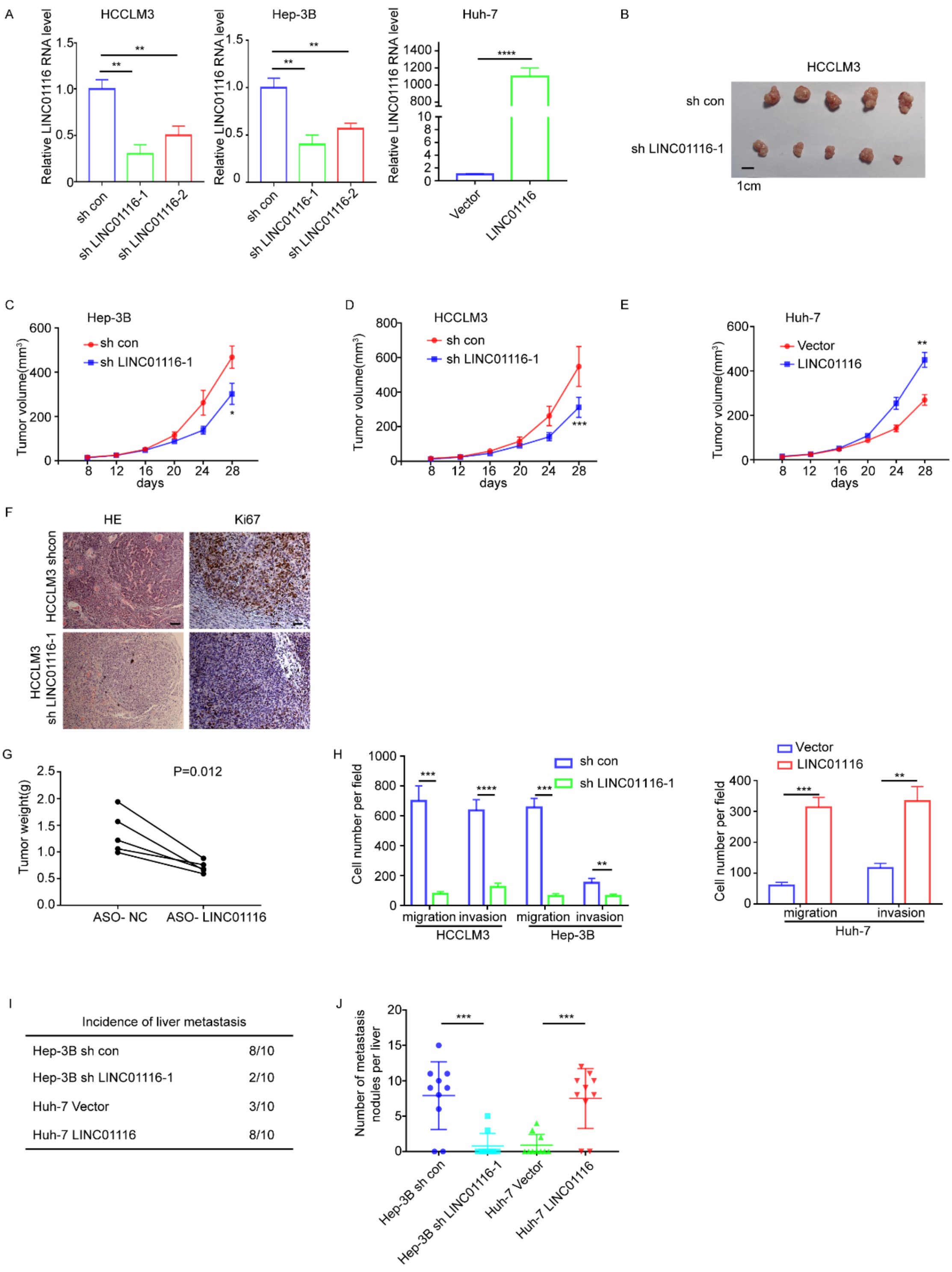

Figure S2 Effects of LINC01116 in HCC progression

A. qRT-PCR detecting the expression of LINC01116 in the indicated cells. **B.** Representative pictures of the tumors from nude mice injected with the indicated cells (n=5 mice/group). **C-E.** Changes in tumor volume of various groups. **F.** Representative

images of hematoxylin and eosin staining (Scale bar 100μm) and Ki67 staining of subcutaneous xenograft model mice (Scale bar 500μm). **G.** The tumor weight of each group (n=5 mice/group). **H.** Statistical analysis of wound healing assays. **I.** Statistical analysis of the incidence of liver metastasis. **J.** Statistical analysis of the number of metastasis nodules. *$p < 0.05$; **$p < 0.01$; ***$p < 0.001$; ****$p < 0.0001$, based on Student t test.

Fig. S3

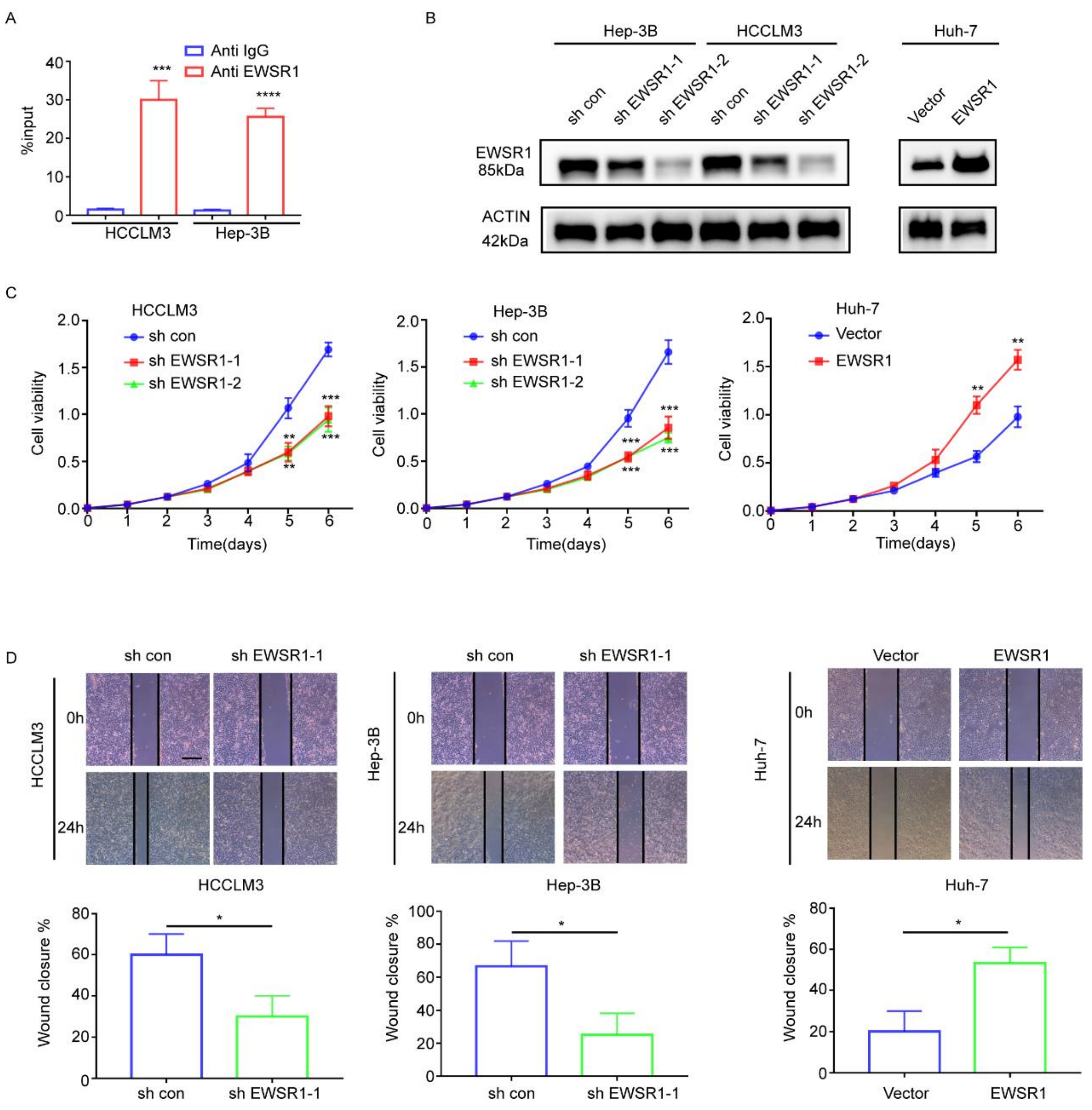

Figure S3 EWSR1 promotes the biological behavior of HCC cells

A. RIP and qRT-PCR assays showed the interaction between EWSR1 and LINC01116. **B.** WB assays detected the EWSR1 expression in the indicated cells. **C.** The proliferation ability of HCC cell lines after knockdown or overexpression of EWSR1 detected by CCK8 assays. **D.** Representative images (scale bar 400 μm) and statistical analysis of wound healing assays. *p < 0.05, **p < 0.01; ***p < 0.001 based on Student t test.

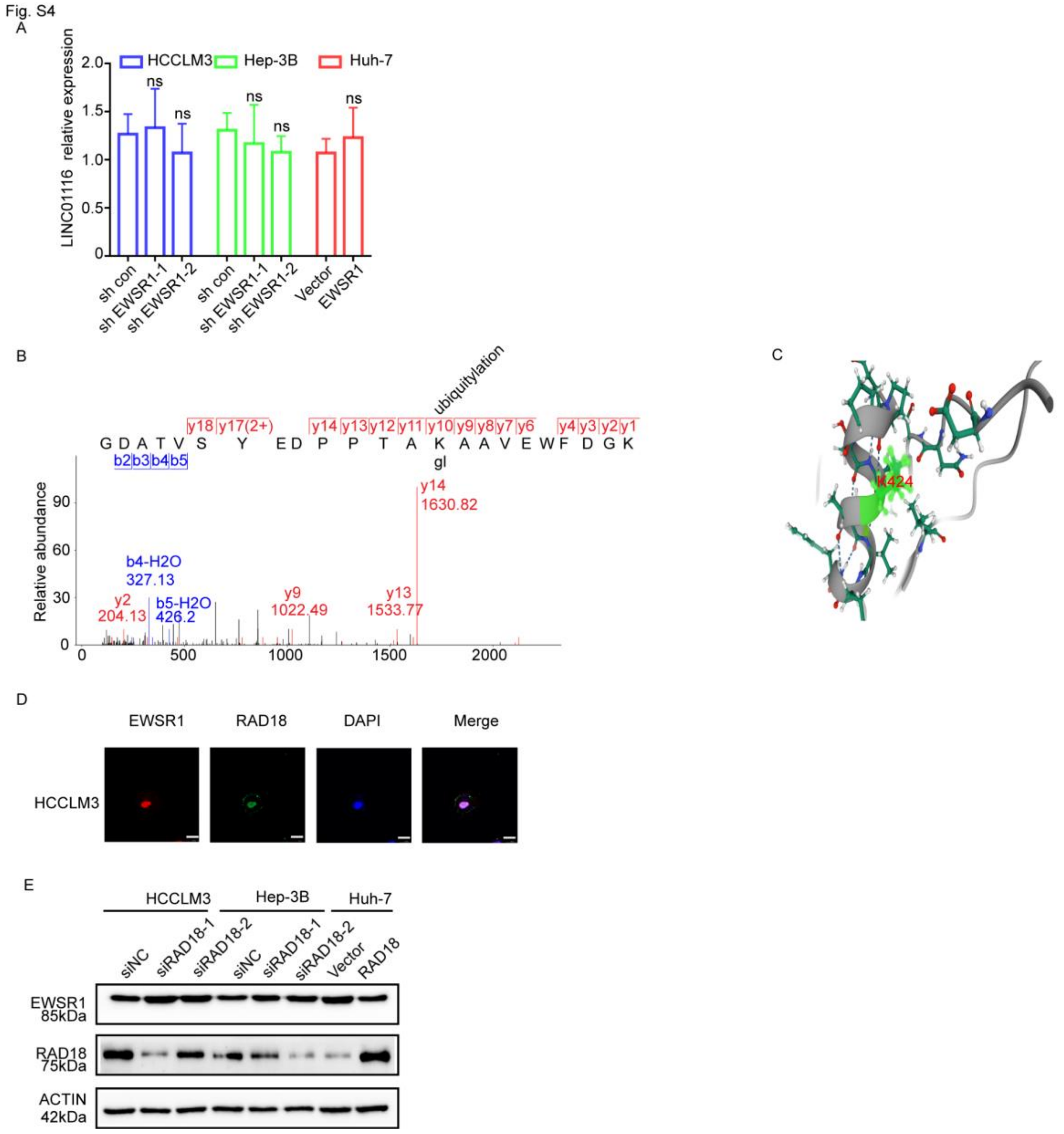

Figure S4 LINC01116 could stabilize EWSR1 by blocking the binding of EWSR1 to RAD18

A. qRT-PCR showed the relative expression of LINC01116 in the indicated cells. **B.** The ubiquitin modification site of EWSR1 was identified by LC-MS/MS. **C.** Crystal structure of EWSR1 protein with K424. **D.** Immunofluorescence showed that EWSR1 colocalized with RAD18 in HCCLM3 cell. Scale bar, 20 μm. **E.** WB assays showed the expression of EWSR1 and RAD18 in the indicated cells.

Fig. S5

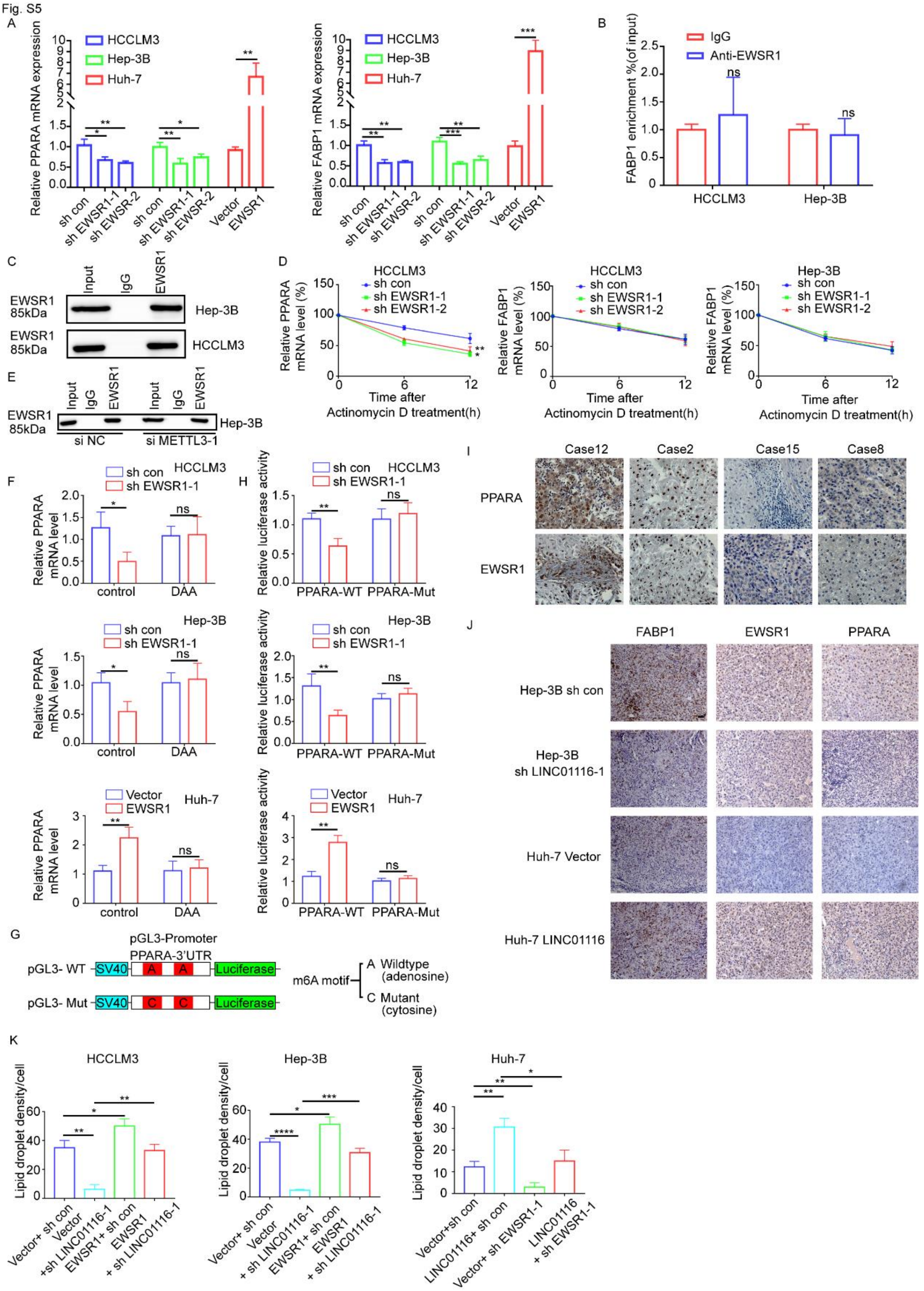
A
Relative PPARA mRNA expression
HCCLM3
Hep-3B
Huh-7
sh con
sh EWSR1-1
sh EWSR-2
Vector
EWSR1
Relative FABP1 mRNA expression
B
FABP1 enrichment %(of input)
IgG
Anti-EWSR1
ns
HCCLM3
Hep-3B
C
Input
IgG
EWSR1
EWSR1 85kDa
Hep-3B
HCCLM3
D
Relative PPARA mRNA level (%)
Relative FABP1 mRNA level (%)
sh EWSR1-2
Time after Actinomycin D treatment(h)
E
si NC
si METTL3-1
F
Relative PPARA mRNA level
control
DAA
H
Relative luciferase activity
PPARA-WT
PPARA-Mut
I
Case12
Case2
Case15
Case8
PPARA
EWSR1
J
FABP1
Hep-3B sh con
Hep-3B sh LINC01116-1
Huh-7 Vector
Huh-7 LINC01116
G
pGL3-Promoter
PPARA-3'UTR
pGL3- WT
pGL3- Mut
SV40
Luciferase
m6A motif
A Wildtype (adenosine)
C Mutant (cytosine)
K
Lipid droplet density/cell
Vector+ sh con
Vector +sh LINC01116-1
EWSR1+ sh con
EWSR1 + sh LINC01116-1
LINC01116+ sh con
Vector+ sh EWSR1-1
LINC01116 + sh EWSR1-1

Fig. S5 Continue

L

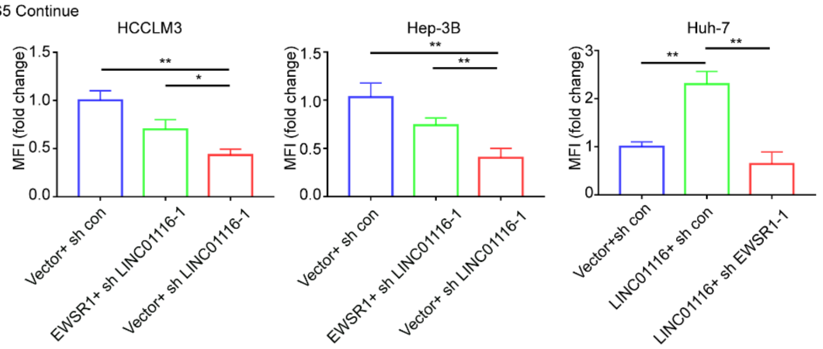

M

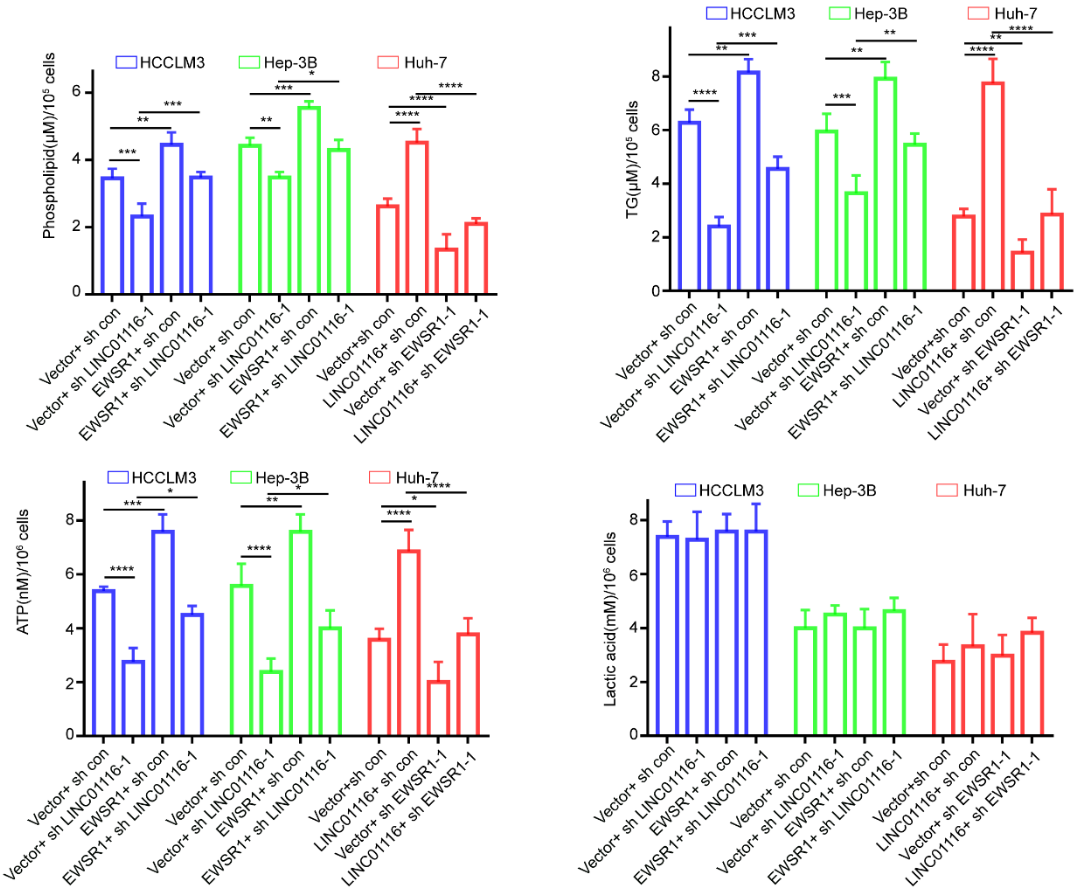

N

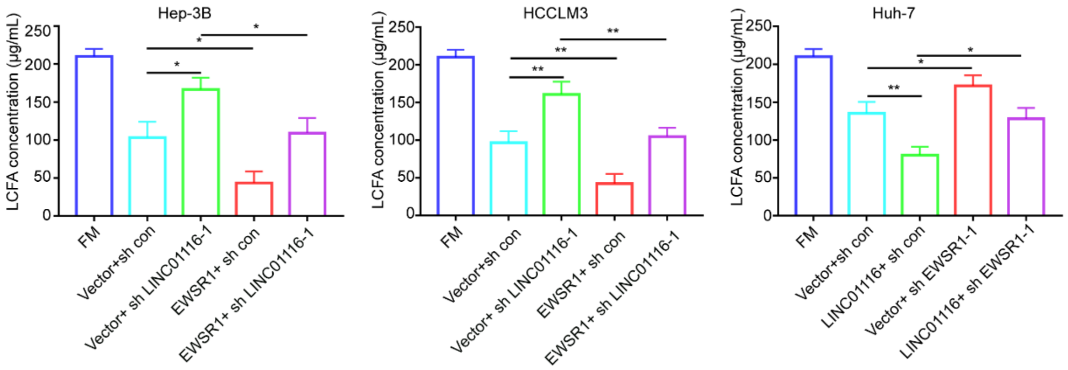

Fig. S5 Continue

O

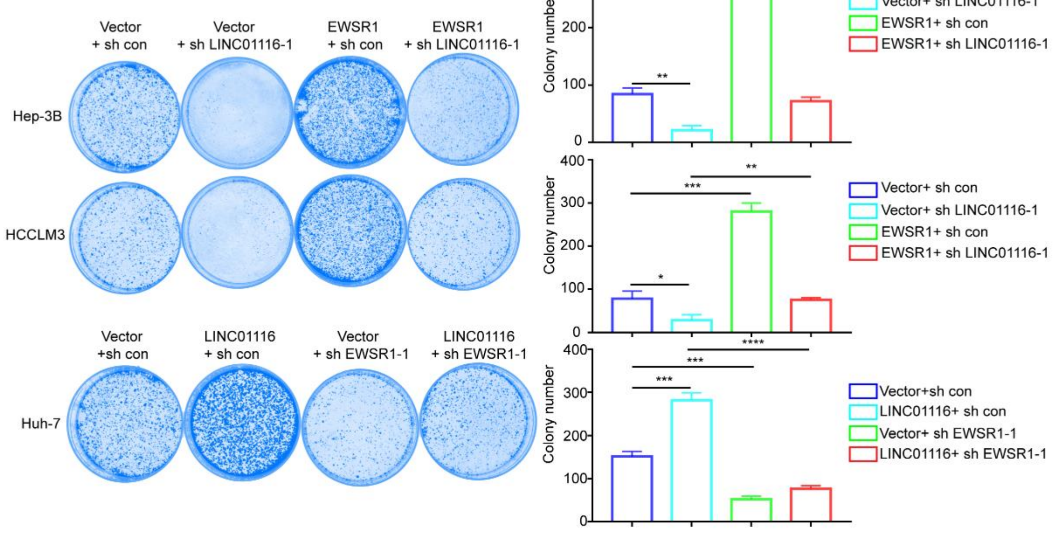

P

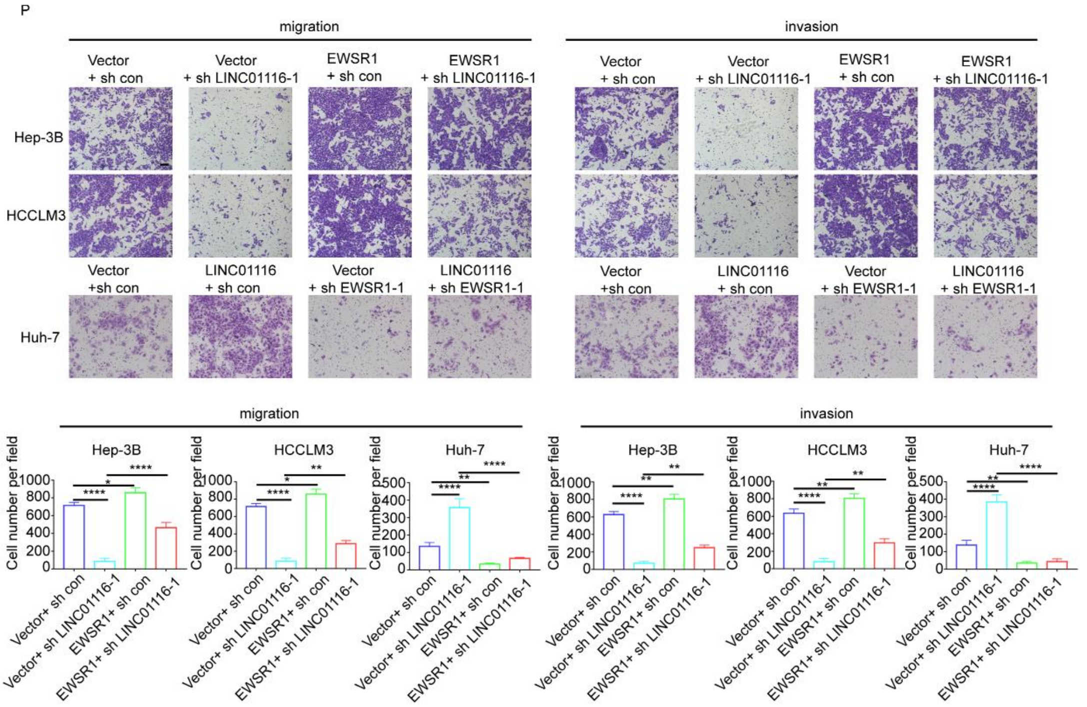

Fig. S5 Continue

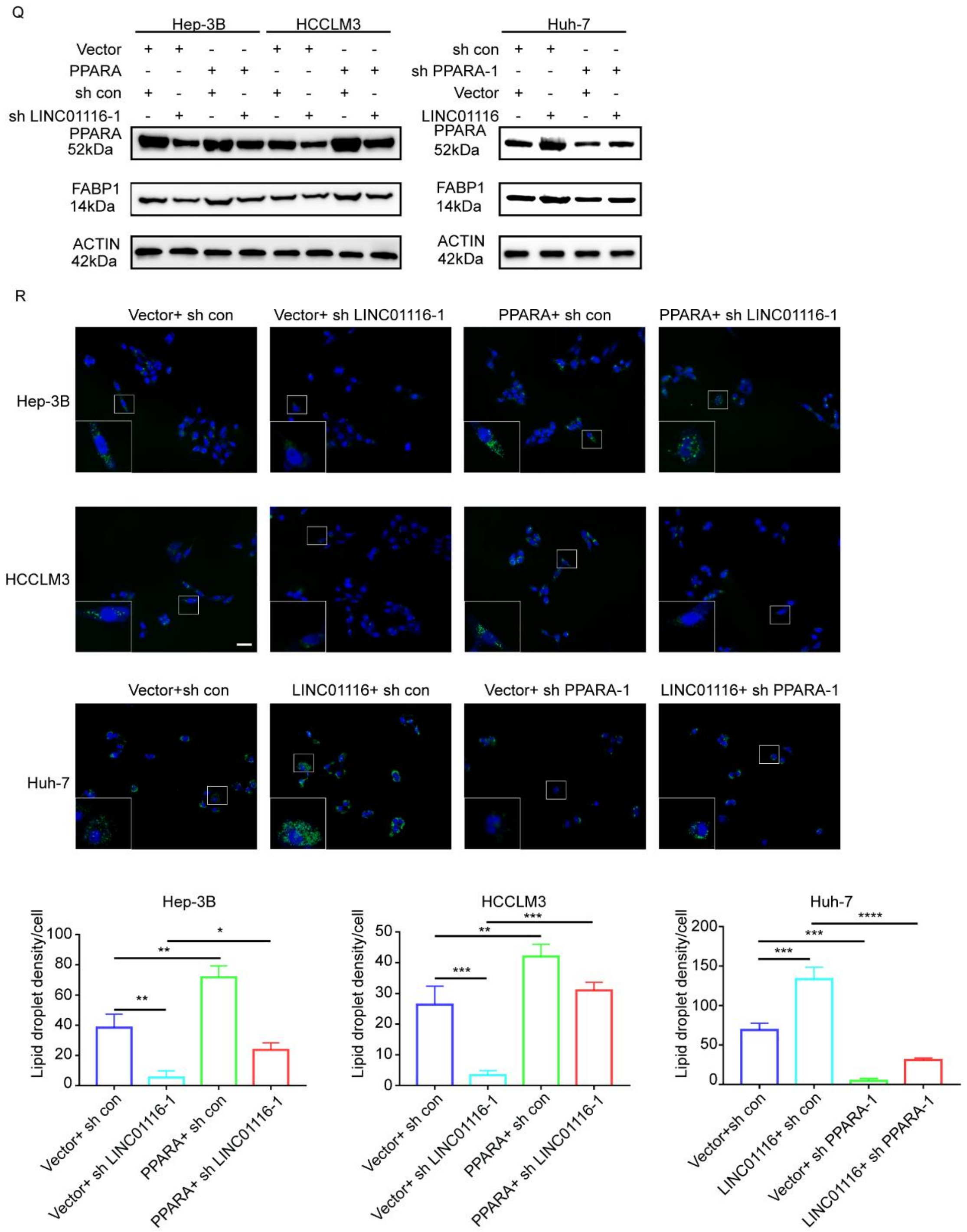

Fig. S5 Continue

S

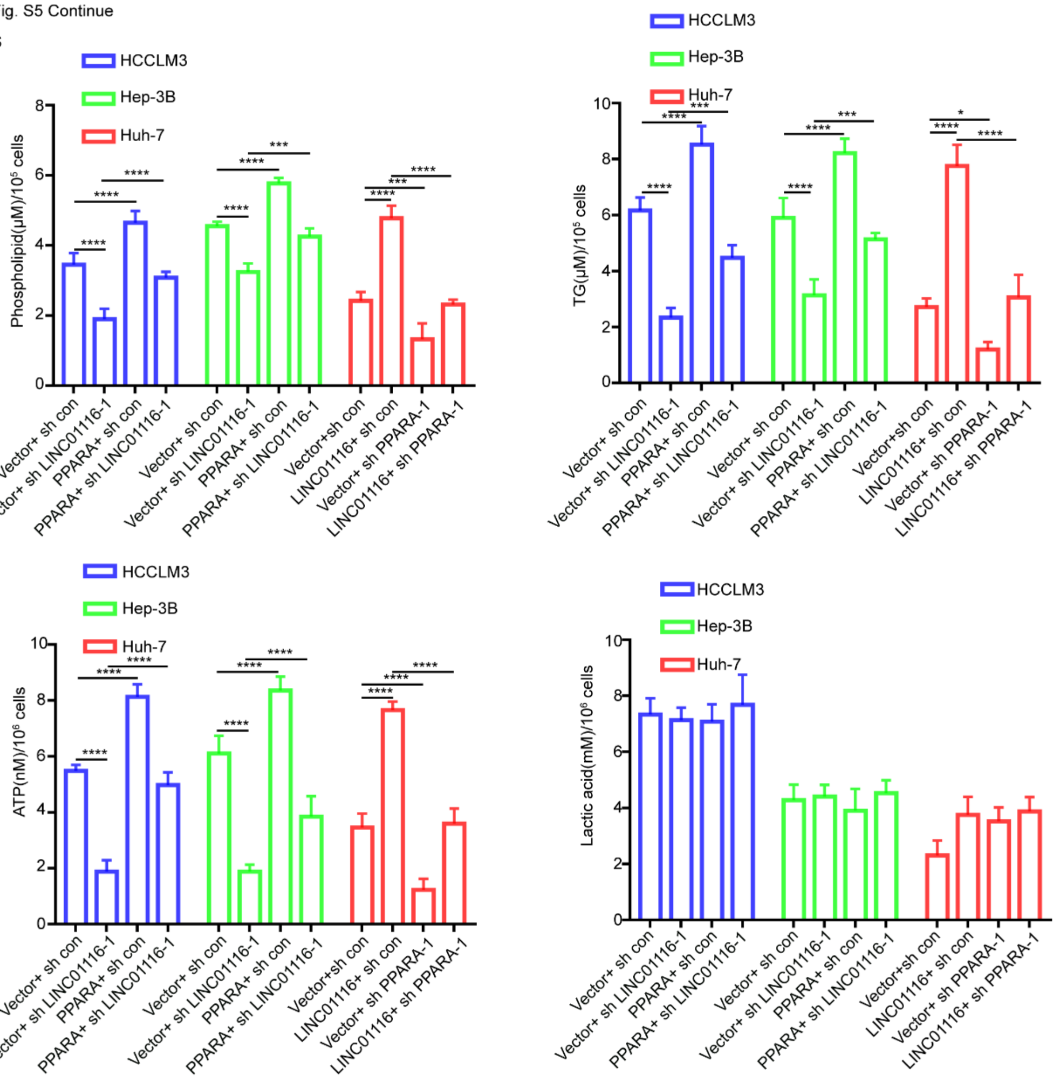

T

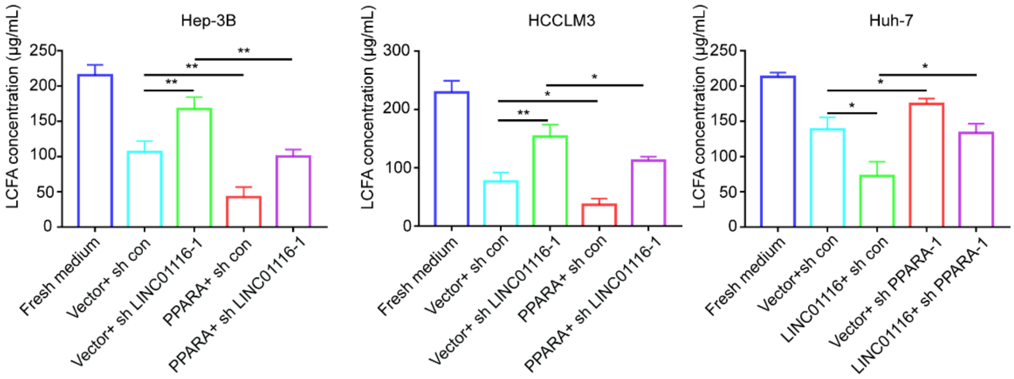

Fig. S5 Continue

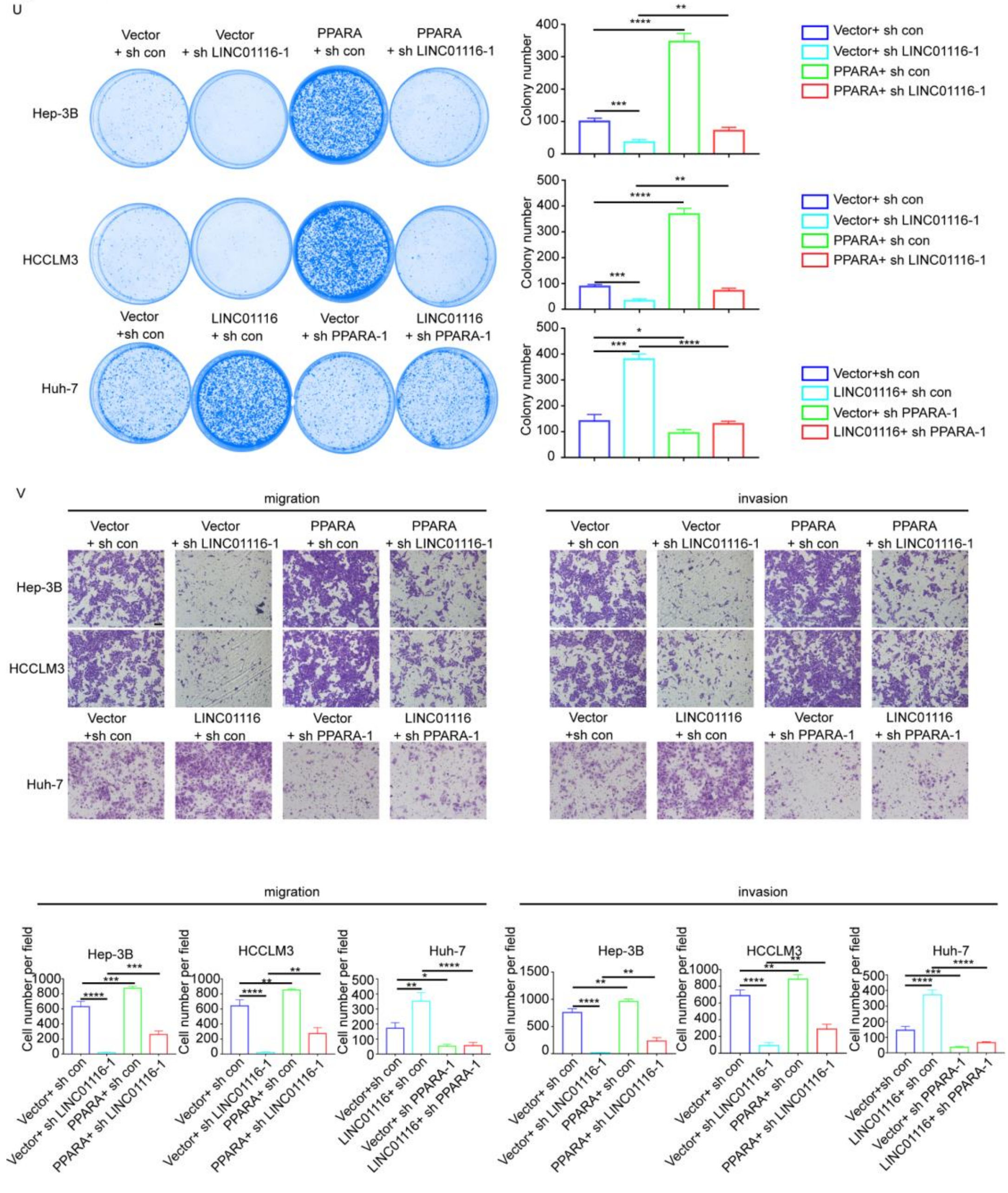

Figure S5 LINC01116 exhibits cancer-promoting properties by altering lipid metabolism through EWSR1/PPARA/FABP1 signal pathway.

A. qRT-PCR showed the relative expression of PPARA and FABP1 mRNA in the indicated cells. **B.** RIP-qRT-PCR showed the FABP1 enrichment in HCCLM3 and Hep-

3B cells. **C.** Immunoblot analysis of input and anti-EWSR1 immunoprecipitates (IPs) derived from HCCLM3 and Hep-3B cells. **D.** qRT–PCR assays showed the mRNA levels of PPARA and FABP1 in indicated cell lines treated with actinomycin D (2 μg/ml) at the indicated time points. **E.** Immunoblot analysis of input and anti-EWSR1 immunoprecipitates (IPs) derived from Hep-3B cells transfected with si NC and si METTL3-1. **F.** Relative PPARA mRNA level of the indicated group. **G.** Graphical explanation for construction of luciferase reporters. The wild-type (full-length) or mutant (m^6A motif mutated) sequence of PPARA–3'UTR was inserted into a pGL3 vector between SV40 and luciferase elements. **H.** Relative luciferase activity for the indicated group. **I.** Typical images of IHC staining analysis of PPARA and EWSR1 expression in 25 HCC human tissues. Scale bar 20μm. **J.** Typical images of IHC staining analysis of FABP1, PPARA and EWSR1 expression in the indicated group. Scale bar 20μm. **K.** Statistics of neutral lipid content detected by immunofluorescence in indicated cells. **L.** Graphs showed MFI values of various groups. **M.** The intracellular levels of PLs, TGs, ATP and lactic acids in the indicated cells were measured with reagent kits. **N.** The content of LCFA in the culture medium supernatant was analyzed by ELISA. **O.** Representative images and statistics of colony-formation assays of the indicated cells. **P.** Representative images and statistics of Transwell migration and Matrigel invasion assays of the indicated cells. **Q.** WB showed the expression of PPARA and FABP1 in the indicated cells. **R.** Representative images and statistics of neutral lipid content detected by immunofluorescence in indicated cells. White boxes showed the representative pictures. Scale bar 50μm. **S.** The intracellular levels of PLs,

TGs, ATP and lactic acids in the indicated cells were measured with reagent kits. **T.** The content of LCFA in the culture medium supernatant was analyzed by ELISA. **U.** Representative images and statistics of colony-formation assays of the indicated cells. **V.** Representative images and statistics of Transwell migration and Matrigel invasion assays of the indicated cells. The data are presented as the mean ± SD. *$p < 0.05$; **$p < 0.01$; ***$p < 0.001$, ****$p < 0.0001$, based on Student t test.

Fig. S6

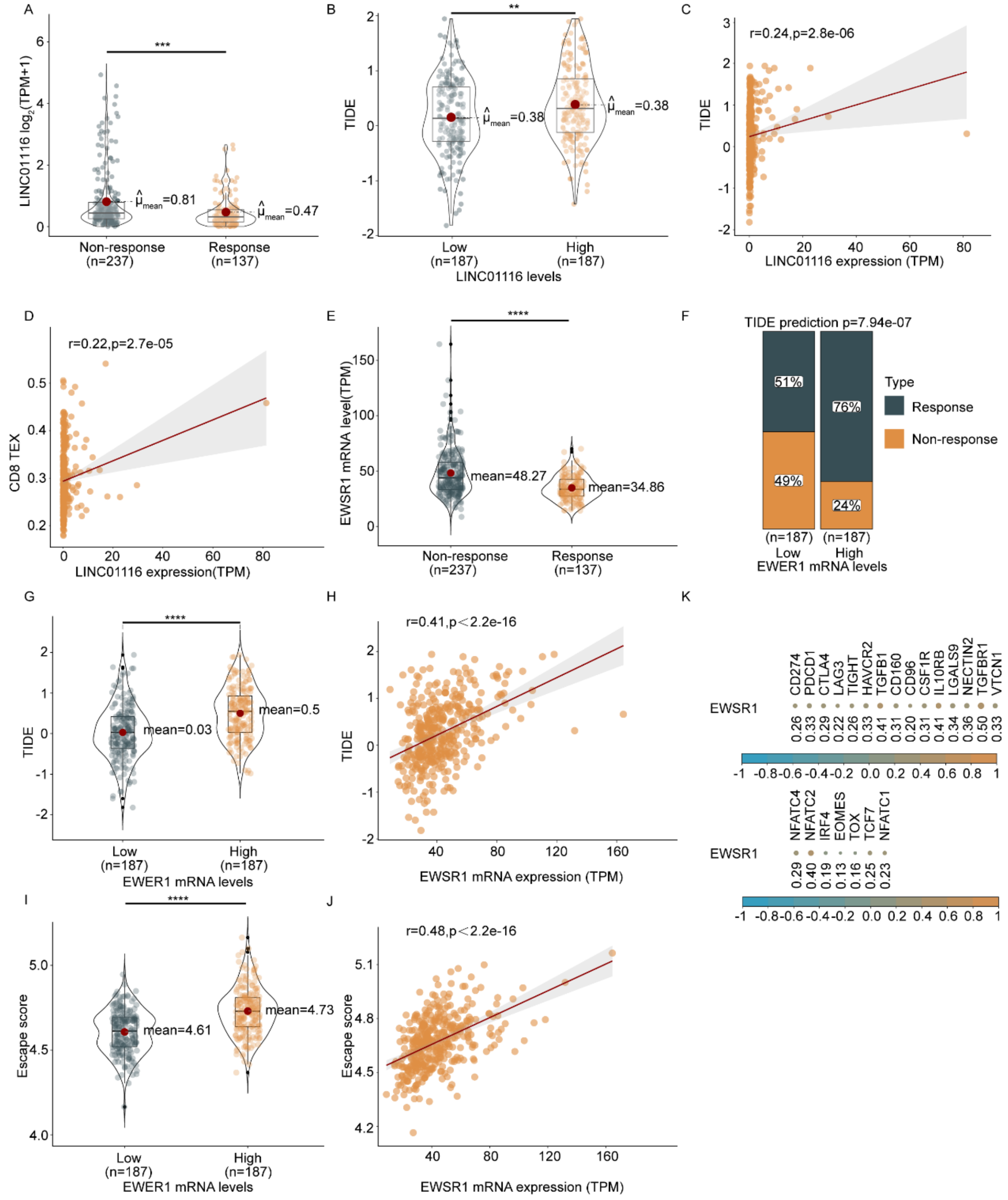
A
LINC01116 log2(TPM+1)
***
μ̂mean=0.81
μ̂mean=0.47
Non-response (n=237)
Response (n=137)
B
TIDE
**
μ̂mean=0.38
μ̂mean=0.38
Low (n=187)
High (n=187)
LINC01116 levels
C
r=0.24,p=2.8e-06
TIDE
LINC01116 expression (TPM)
D
r=0.22,p=2.7e-05
CD8 TEX
LINC01116 expression(TPM)
E
EWSR1 mRNA level(TPM)
****
mean=48.27
mean=34.86
Non-response (n=237)
Response (n=137)
F
TIDE prediction p=7.94e-07
51%
49%
76%
24%
Type
Response
Non-response
(n=187) Low
(n=187) High
EWER1 mRNA levels
G
TIDE
****
mean=0.03
mean=0.5
Low (n=187)
High (n=187)
EWER1 mRNA levels
H
r=0.41,p<2.2e-16
TIDE
EWSR1 mRNA expression (TPM)
K
CD274 PDCD1 CTLA4 LAG3 TIGHT HAVCR2 TGFB1 CD160 CD96 CSF1R IL10RB LGALS9 NECTIN2 TGFBR1 VTCN1
EWSR1
0.26 0.33 0.29 0.22 0.26 0.33 0.41 0.31 0.20 0.31 0.41 0.34 0.36 0.50 0.33
-1 -0.8 -0.6 -0.4 -0.2 0.0 0.2 0.4 0.6 0.8 1
NFATC4 NFATC2 IRF4 EOMES TOX TCF7 NFATC1
EWSR1
0.29 0.40 0.19 0.13 0.16 0.25 0.23
-1 -0.8 -0.6 -0.4 -0.2 0.0 0.2 0.4 0.6 0.8 1
I
Escape score
****
mean=4.61
mean=4.73
Low (n=187)
High (n=187)
EWER1 mRNA levels
J
r=0.48,p<2.2e-16
Escape score
EWSR1 mRNA expression (TPM)

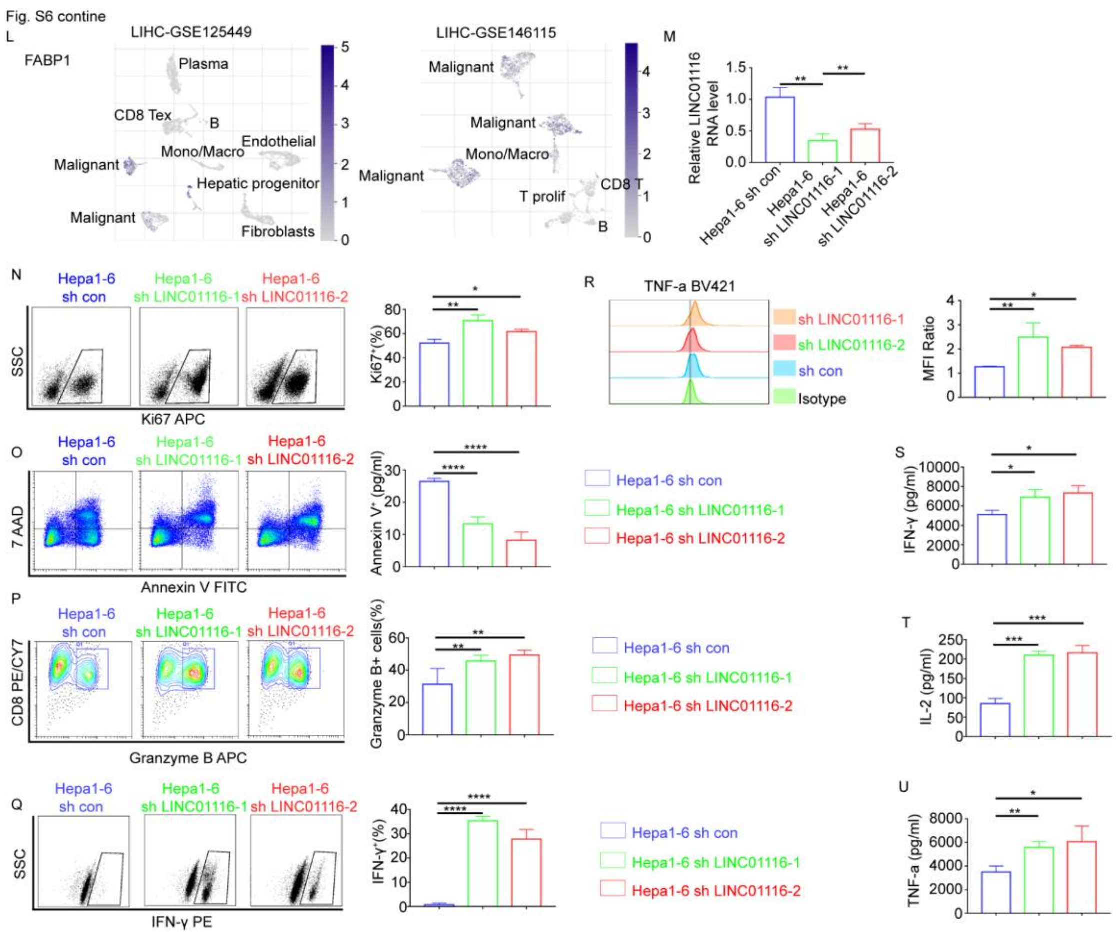

Figure S6 LINC01116 is associated with immunotherapy insensitivity and T-cell exhausted

A. Based on TIDE prediction, the differential expression of LINC01116 in response and non-response groups was analyzed. **B.** Tide score of the indicated groups. **C.** The correlation between LINC01116 expression and TIDE score was analyzed. **D.** Correlation analysis between LINC01116 expression and CD8_TEX_HCC score calculated based on ssGSEA algorithm. **E.** Based on TIDE prediction, the differential expression of EWSR1 in response and non-reponse groups was analyzed. **F.** Proportion of patients responded to immunotherapy based on the median value of EWSR1. **G.** Tide score of the indicated groups. **H.** Correlation analysis between EWSR1 mRNA level

and Tide score. **I.** The difference of IPS score between the high and low expression groups based on the median value of EWSR1. **J.** Correlation analysis between EWSR1 expression and escape score. **K.** Correlation analysis between the mRNA expression of EWSR1 and immune checkpoints or the expression of transcription factors leading to T cell exhaustion. **L.** Expression of FABP1 mRNA in various types of cells in the TISCH database. **M.** Relative LINC01116 level for the indicated group. **N.** Representative flow plots and quantification of Ki67$^+$ T cells for the indicated group (n=3/group). **O.** Representative flow plots and quantification of Annexin V$^+$ T cells for the indicated group (n=3/group). **P.** Representative flow plots and quantification of Granzyme B$^+$ T cells for the indicated group (n=3/group). **Q.** Representative flow plots and quantification of IFN-γ$^+$ T cells for the indicated group (n=3/group). **R.** Representative histograms and quantification of activated T cells for the indicated group (n=3/group). **S-U.** The amount of cytokines was measured by ELISA. The data are presented as the mean ± SD. *p < 0.05; **p < 0.01; ***p < 0.001, ****p < 0.0001, based on Student t test.

Fig.S7

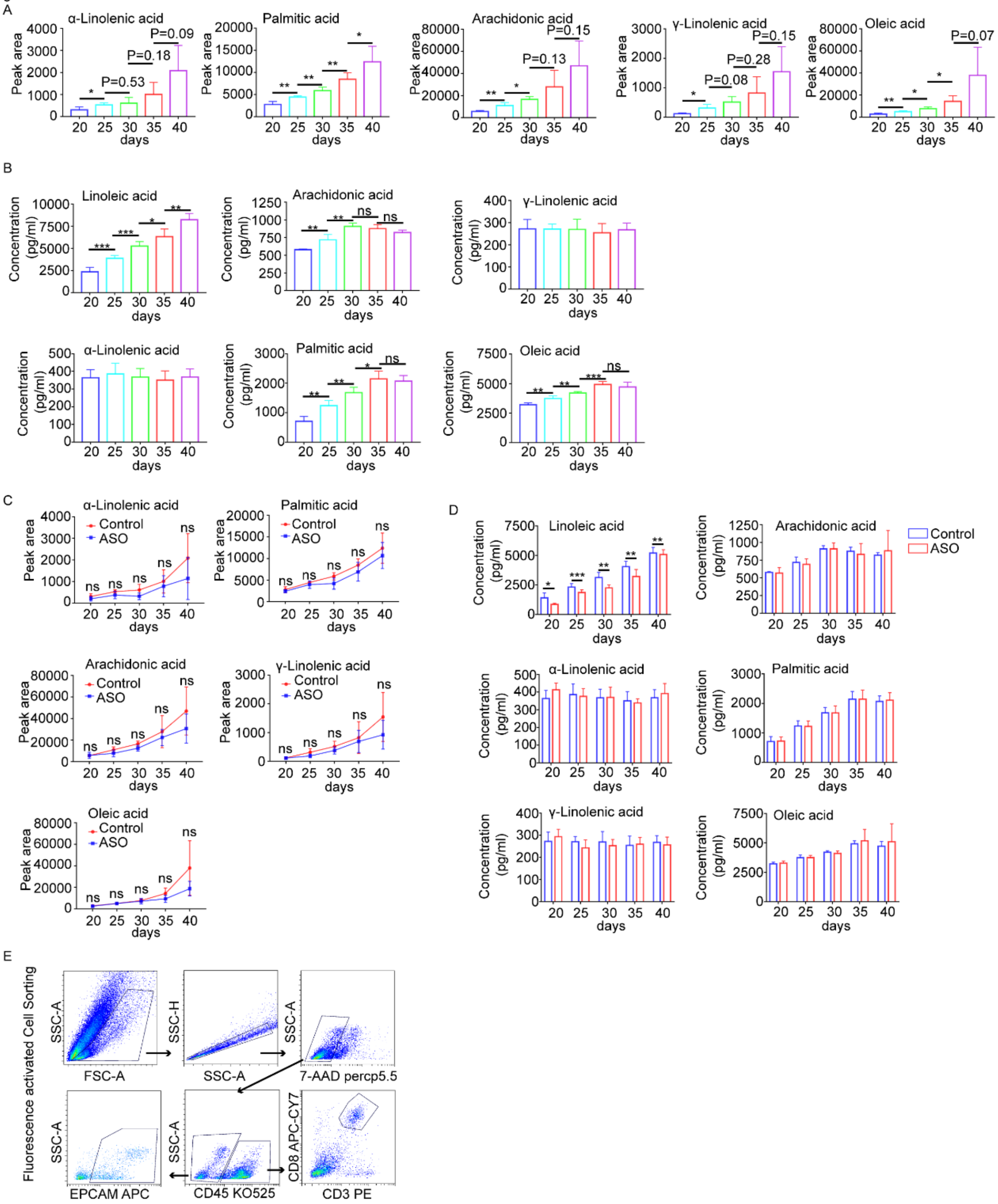

A
α-Linolenic acid
Palmitic acid
Arachidonic acid
γ-Linolenic acid
Oleic acid
Peak area
days
P=0.09
P=0.18
P=0.53
P=0.15
P=0.13
P=0.28
P=0.08
P=0.07
B
Linoleic acid
Arachidonic acid
γ-Linolenic acid
α-Linolenic acid
Palmitic acid
Oleic acid
Concentration (pg/ml)
days
C
Control
ASO
D
Control
ASO
E
Fluorescence activated Cell Sorting
FSC-A
SSC-A
SSC-H
7-AAD percp5.5
EPCAM APC
CD45 KO525
CD8 APC-CY7
CD3 PE

Fig. S7 contine

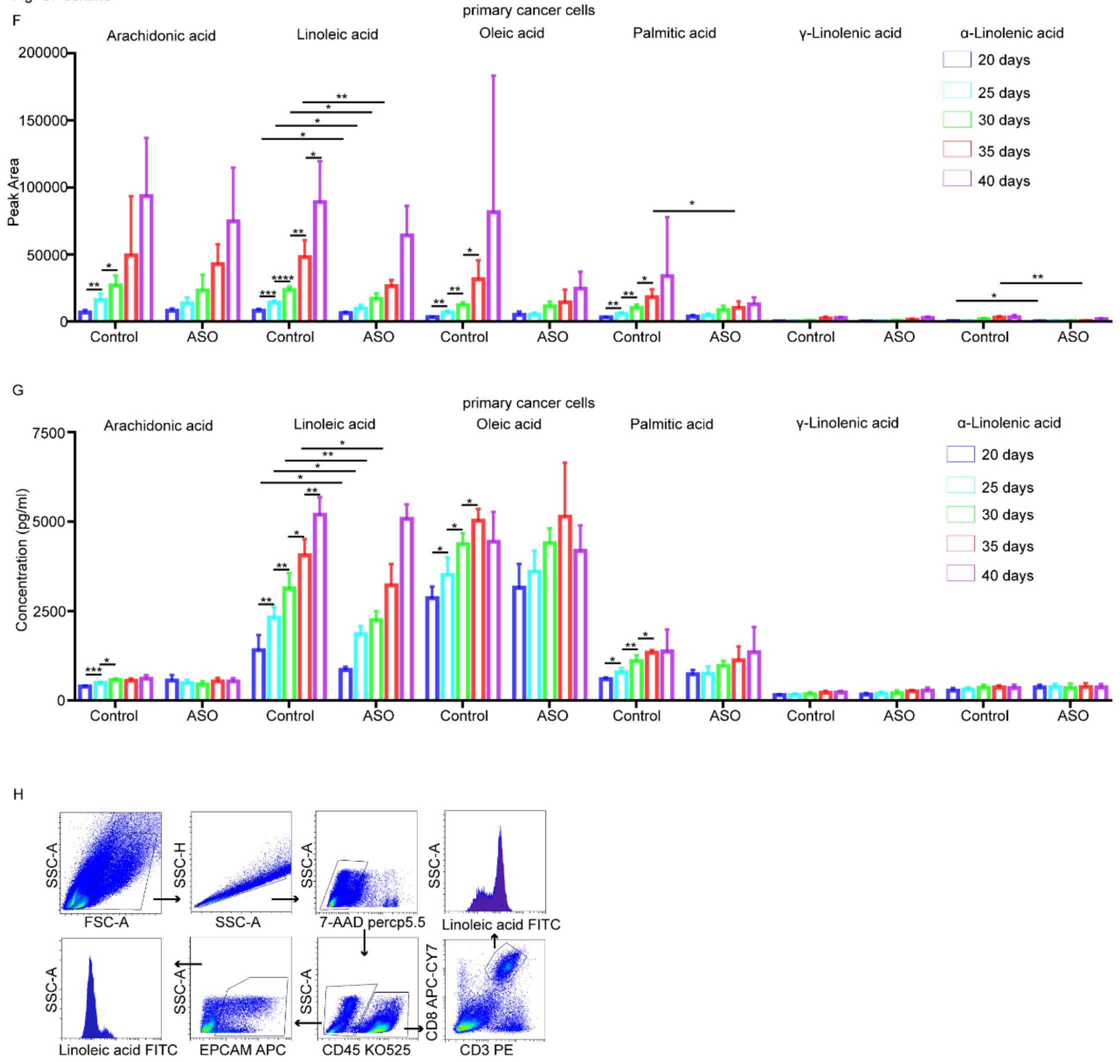

Figure S7 Changes in typical fatty acids in tumour progression

A. LC-MS/MS analysis for fatty acids of the tumor tissues at the different time points (n=5/group). **B.** Fatty acids of the tumor tissues were measured by ELISA. **C.** LC-MS/MS analysis for typical fatty acids of the indicated group at the different time points (n=5/group). **D.** Typical fatty acids content of the indicated group at the different time points were measured by ELISA. **E.** Gating strategy for primary cancer cells ($CD45^-$ $EPCAM^+$) and $CD8^+$ T cells ($CD45^+CD3^+CD8^+$). **F.** LC-MS/MS analysis for typical fatty acids of the indicated group at the different time points (n=5/group). **G.** Typical

fatty acids content of the indicated group at the different time points were measured by ELISA. **H.** Gating strategy for $FITC^+$ cells. The data are presented as the mean ± SD. $*p < 0.05$; $**p < 0.01$; $***p < 0.001$, $****p < 0.0001$, based on Student t test.

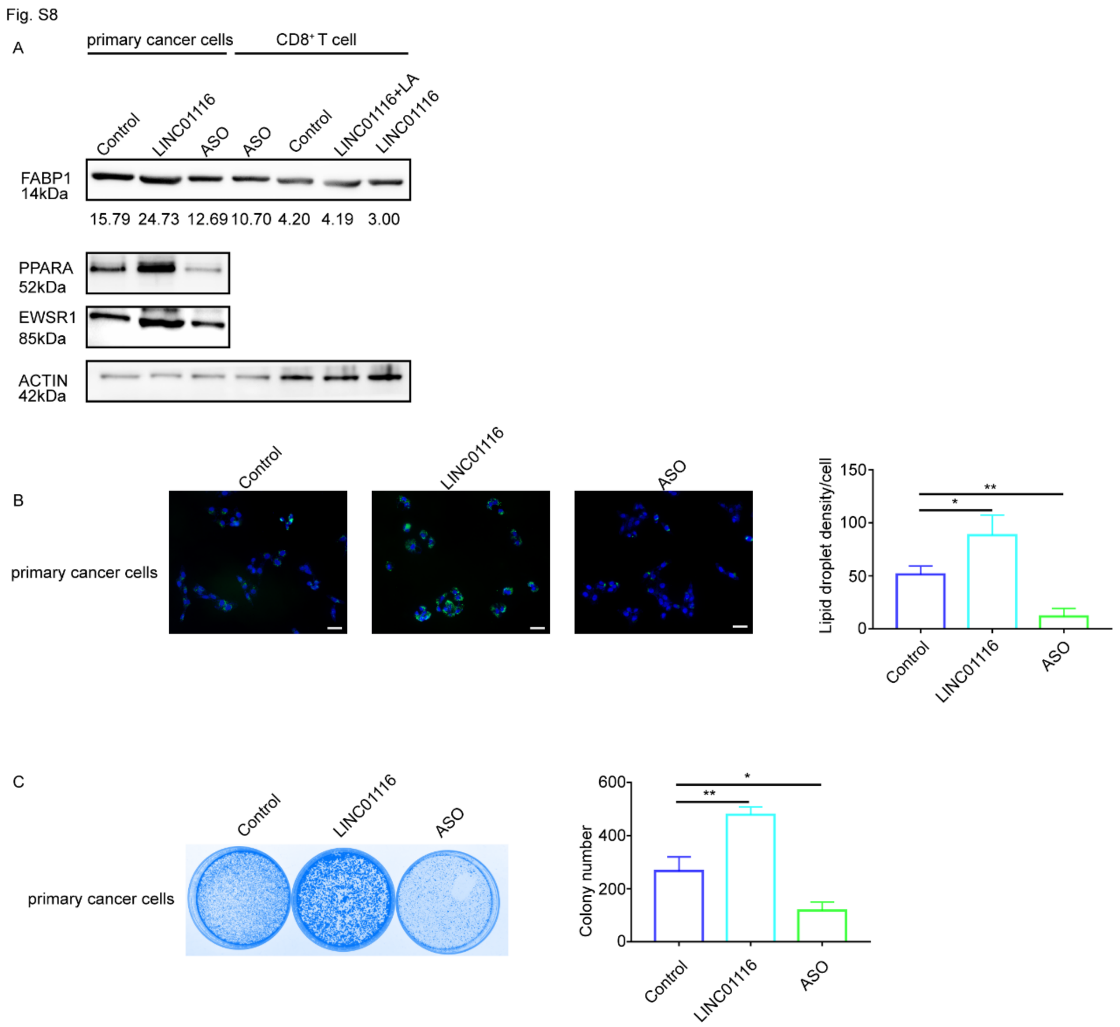

Figure S8 The role of LINC01116 in the primary cancer cells

A. Expression of FABP1, PPARA and EWSR1 in the indicated group detected by WB. **B.** Immunofluorescence showed the density of lipid droplets in the indicated cells and statistical analyses were showed. Scale bars 50 μm. **C.** Representative images of colony formation assays and colony counts. The data are presented as the mean ± SD. *p < 0.05; **p < 0.01, based on Student t test.

Fig. S9

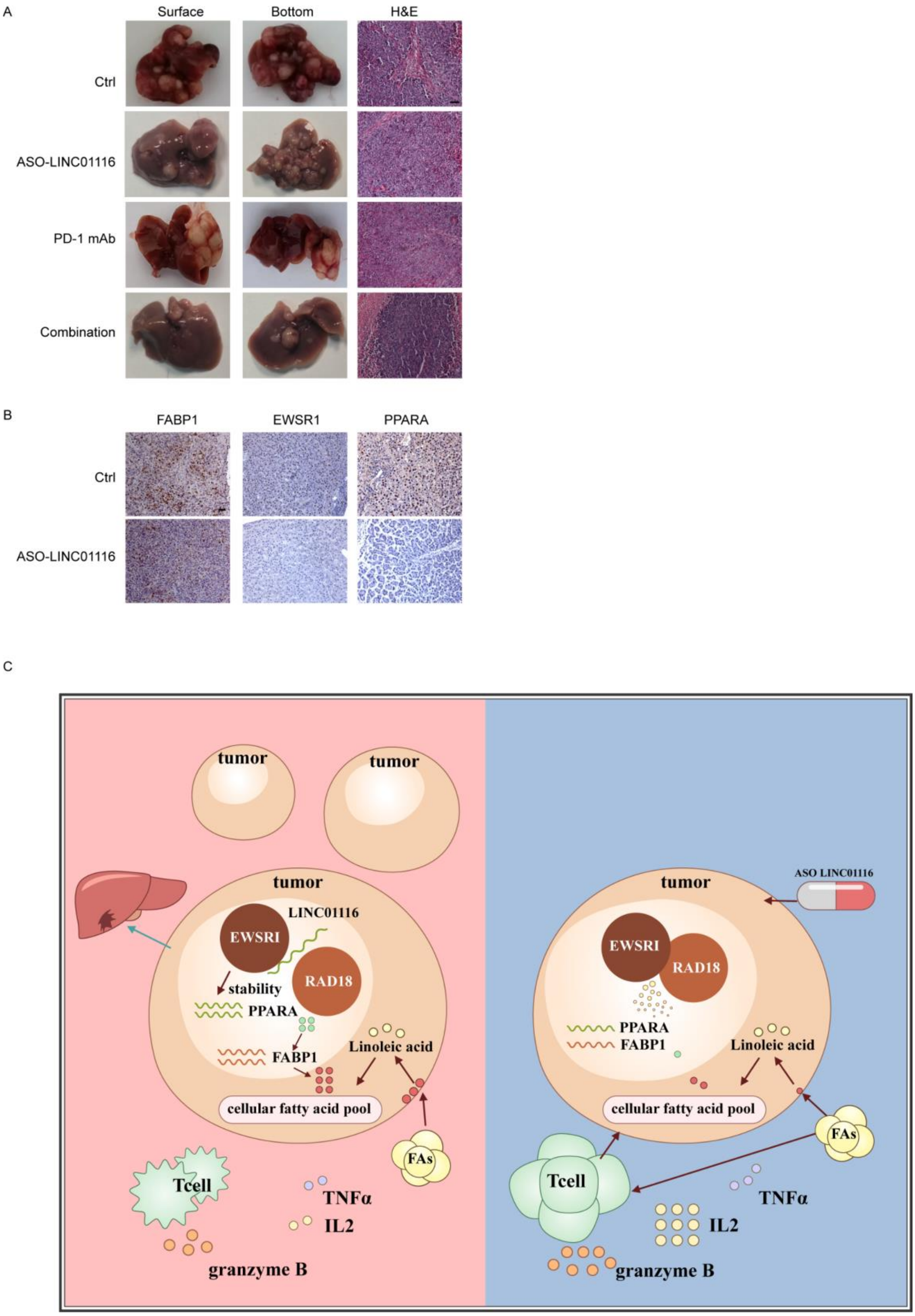

Figure S9 Inhibition of LINC01116 can enhance the sensitivity to immunotherapy

A. Representative images of specimens, hematoxylin and eosin staining. Scale bars 50

μm. **B.** Typical images of IHC staining analysis of PPARA, FABP1 and EWSR1 expression. **C.** Schematic of the result.

Table S1. Patient Characteristic of Cohort 2

	Low expression of LINC01116 (n=25)	High expression of LINC01116 (n=25)	P value
Age（≤60/＞60）	20/5	22/3	N.S.
Gender(F/M)	12/13	11/14	N.S.
TNM (I-II/III-IV)	14/11	6/19	0.0209
Vascular invasion (Yes/No)	7/18	15/10	0.0227
Tumor size (≤5cm/＞5cm)	15/10	7/18	0.0227

Table S2. Summary of primary antibodies used in western Blot, immunochemistry, immunofluorescence, co-immunoprecipitation, mIHC and flow cytometry

**Western blot materials**		
PPARA	Proteintech	15540-1-AP
FABP1	Proteintech	13626-1-AP
EWSR1	CST	#11910
β-Actin	Sigma	A2228
DYKDDDK	CST	#14793
DYKDDDK	CST	#8146
RAD18	Proteintech	18333-1-AP
HA	Abcam	ab1424
HA	CST	#3724

**IF materials**		
EWSR1	Abcam	ab133288
RAD18	Proteintech	18333-1-AP

**IP materials**		
EWSR1	CST	#11910
DYKDDDK	CST	#14793

DYKDDDK	CST	#8146

**Flow cytometry materials**		
APC-Granzyme B	Biolegend	372203
Live	Biolegend	423105
BB515-CD8	Biolegend	100705
BV510-CD45	Biolegend	103137
BB700-CD3	BD Biosciences	566494
AF700-CD279	BD Biosciences	744548
BV786-TIM3	BD Biosciences	566346
ANNEXIN V/7-AAD	Biolegend	640922
BV421-TNF-a	Biolegend	506327
APC-Ki67	Biolegend	561126
PE/CY7-Ki67	Biolegend	151217
PE-IFN-γ	Biolegend	505808
APC/CY7-CD3	Biolegend	100222
PE/CY7-CD8	Biolegend	100721
PE-CD3	Biolegend	100205
APC/CY7-CD8	Biolegend	100765
APC-EPCAM	Biolegend	118213

**IHC materials**

EWSR1	Abcam	ab133288
PPARA	Abcam	ab233078
PPARA	Proteintech	66826-1-Ig
FABP1	Abcam	ab171739
Granzyme B	CST	#46890
CD8	CST	#98941
CD8	CST	#85336
CD3	CST	#85061
CD3	CST	#78588
Ki67	CST	34330SF
PD-1	CST	#84651
PD-1	CST	#86163

Table S3. Primers and target sequences in this study

qPCR Primers		Sequence
EWSR1	forward	5'- AAAGGCGATGCCACAGTGTCCT -3'
	reverse	5'- TCATTGGAGGCTTCTTCCGAGC -3'
ACTB	forward	5'- CTCGCCTTTGCCGATCC -3'
	reverse	5'- GAATCCTTCTGACCCATGCC -3'
FABP1	forward	5'- GGAGGAATGTGAGCTGGAGACA -3'
	reverse	5'- TATGTCGCCGTTGAGTTCGGTC -3'
PPARA	forward	5'- TCGGCGAGGATAGTTCTGGAAG -3'
	reverse	5'- GACCACAGGATAAGTCACCGAG -3'
LINC01116(H)	forward	5'- CCCGAGTACCTGACTGAGGA -3'
	reverse	5'- TCCAAGCAGGGAGGTCAAAC -3'
LINC01116(M)	forward	5'- GCCCATGTTACAGGGAGTCT -3'
	reverse	5'- TCAGCTCAAACGTCCTTCCT -3
DNA probes		
Sense1		GCTCGTCTTCAGCAAAGCGA
Sense2		TTTTCACTTGAAATATTGAA
Sense3		GCAAGTCCTTTTCACTTATT
Anti-sense		TCGCTTTGCTGAAGACGAGC

shRNA		Target sequence
LINC01116 sh1	H	GCAGTGTATTAGAAGACAACT

LINC01116 sh2	H	GAATAGAAATGCTAACCTACC
LINC01116 sh1	M	GAAAGTTATGTTCTTCCAAGG
LINC01116 sh2	M	GTGTGTTAATTGGAAGTTAGT
EWSR1 sh1	H	GACCGCCTATGCAACTTCTTA
EWSR1 sh2	H	TACGGGCAGCAGAGTTCATTC
RAD18 si1	H	TGCTTCGAGTATTTCAACATT
RAD18 si2	H	GAACCAAGAAACAAGCGTAAT
PPARA sh1	H	GAACAGAAACAAATGCCAGTA
PPARA sh2	H	GTAGCGTATGGAAATGGGTTT
METTL3 si1	H	GACGAATTATCAATAAACACA
METTL3 si2	H	AGCTACAGATCCTGAGTTAGA
